# Metabolic Labeling of Phosphatidylethanolamine Lipids in Cells Using Clickable Ethanolamine Analogs

**DOI:** 10.1002/cbic.70501

**Published:** 2026-08-14

**Authors:** Robert J. Maraski, Jinchao Lou, Todd B. Reynolds, Michael D. Best

**Affiliations:** ^1^ Department of Chemistry University of Tennessee Knoxville Tennessee USA; ^2^ Department of Microbiology University of Tennessee Knoxville Tennessee USA

**Keywords:** click chemistry, lipids, metabolic labeling, phosphatidylethanolamine

## Abstract

Phosphatidylethanolamine (PE) is the second most abundant phospholipid in mammalian cells. The cone‐shaped structure of PE positions this lipid to act as a cornerstone of signaling events, such as those involving protein–protein and lipid–protein interactions, as it has been shown to promote conformational changes in both protein and lipid‐based membrane structures. Despite this importance, chemical tools to interrogate the biological activity and trafficking of PE lipids are limited. Herein, we report the development of ethanolamine probes functionalized with clickable tags that are capable of infiltrating native cellular pathways and subsequently producing labeled PE lipids. Derivatization of click‐tagged PE products enabled fluorescence microscopy imaging of these lipids in cells. Interrogation of the efficacy of these probes for biological incorporation was conducted using mass spectrometry and thin‐layer chromatography analysis to identify various species of tagged PE molecules. This approach provides an invaluable step toward metabolic labeling strategies that will aid in the tracking of PE biosynthesis and trafficking pathways in cells.

## Graphical abstract

This work describes the metabolic labeling of phosphatidylethanolamine (PE) lipids using clickable ethanolamine substrate analogs. Post‐derivatization of tagged PE in cells via click chemistry enabled detection using fluorescence microscopy, mass spectrometry, and TLC imaging.

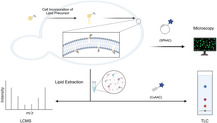

## 1 Introduction

Phosphatidylethanolamine (PE) is a zwitterionic phospholipid that comprises between 15% and 20% of total lipid composition in mammalian cells [1]. This lipid plays crucial roles in biological processes, such as the regulation of autophagy and the formation of nonbilayer lipid structures within both yeast and mammalian cells [2]. Dysregulation of PE has been associated with insulin resistance and skeletal muscle dysfunction, as well as diseases such as cancer and diabetes [3, 4]. Given its essential roles, the ability to effectively label and track PE is crucial in gaining further understanding of the metabolism, trafficking, and biological activities of this lipid.

PE is a downstream product of three pathways in yeast cells. First, it can be produced in the endoplasmic reticulum (ER) via the Kennedy pathway from the precursors of imported ethanolamine (ETA) or ETA‐phosphate derived from phosphorylated sphingoid bases. The other two pathways form PE in the mitochondrion and endosomal membranes from PS through decarboxylation of phosphatidylserine (PS) to PE by PS decarboxylase I and II, respectively [5, 6]. In all three pathways, PE can be subsequently converted to phosphatidylcholine (PC) through PE *N*‐methyltransferase (PEMT) activities of two related enzymes [7]. PC composes up to 50% of total phospholipids in mammalian cells and is crucial in maintaining membrane lipid homeostasis and the production of lipid signaling molecules [8, 9]. PC can be generated from choline and diacylglycerol (DAG) via the Kennedy pathway, but between 10% and 40% of all PC in mammalian cells is generated from PE via PEMT [10]. The multitude of crucial functions coupled with the dysregulation of PC being linked to disease progression indicates that the ability to track PE could also lead to downstream developments elucidating PC metabolism [11].

An increased recognition of the importance of lipids as biological mediators and signaling molecules has given rise to lipid metabolic labeling studies, which involve the introduction of minimally modified substrate‐adjacent biomolecules into cells [12]. Phospholipids provide particularly enticing targets for labeling studies, as they undergo complex interconversions between many different lipid species, are found throughout all parts of the cell, but with individual lipids exhibiting specific subcellular localization, and their dysregulation is linked to various diseases [1]. Strategies such as isotopic labeling were originally employed to elucidate lipid pathways and have been vital for advancing the understanding of lipid activity and metabolism through the subtle introduction of heavy atoms [13]. This enables detection based on mass, while recent developments have provided tools capable of organelle‐specific imaging [14, 16].

An effective alternative approach has involved the installation of bioorthogonal/clickable functional groups onto biologically active substrates, a strategy that has demonstrated the ability to precisely track lipids, nucleotides, and other important biomolecules [17, 18]. In addition to elucidating the localization of these biomolecules, a clickable handle enables the ability to introduce diverse functionality, which has been applied to identify novel proteins based on their activity and provide insight into the metabolic activity of enzymes [19, 20]. In the medical realm, click chemistry has been used to develop new therapeutic scaffolds to enhance drug efficacy [21]. Bioorthogonal handles have been tuned to be unreactive within the confines of native biological systems yet undergo rapid and observable modifications in the presence of their specific click tag partners [22]. Perhaps the most prominent of these click partners are the azide and alkyne, which have been demonstrated to undergo both copper‐catalyzed azide‐alkyne cycloaddition (CuAAC) and strain‐promoted azide‐alkyne cycloaddition (SPAAC) reactions at high rates and yields within complex biological systems [23]. Due to their diminutive size, both the azide and alkyne have been shown to minimally affect metabolic modification of biomolecules into which they have been appended, enabling their utility for furthering our knowledge of glycerophospholipids (GPLs) and other biologically pertinent compounds [24].

Prior work in the field has demonstrated the infiltration of multiple lipid pathways through the introduction of clickable lipid precursors [25, 26]. Salic and coworkers initially showcased this approach by developing clickable choline analogs to produce labeled PC [27, 28]. Inositol‐containing lipids have also been successfully labeled using clickable inositol probes targeting both glycophosphatidylinositol (GPI) anchors [29] and phosphatidylinositol (PI) lipids [30]. Baskin and coworkers have hijacked phospholipase D (PLD) enzymes to incorporate clickable tags into phosphatidic acid (PA) lipids to investigate both PLD and PA dynamics [31]. Our group has also shown the labeling of PS products in cells using clickable serine analogs as substrates [32]. Furthermore, we have also worked to more globally label GPLs using tagged glycerol [33] and caged glycerol‐3‐phosphate [34] probes that can be incorporated into the core scaffold of a variety of GPLs. The current work expands this toolbox to now enable the labeling of important PE lipids using clickable ETA substrate precursors, which is expected to enhance the ability to track the biosynthesis and trafficking of PE in cells.

## 2 Results and Discussion

**FIGURE 1:**
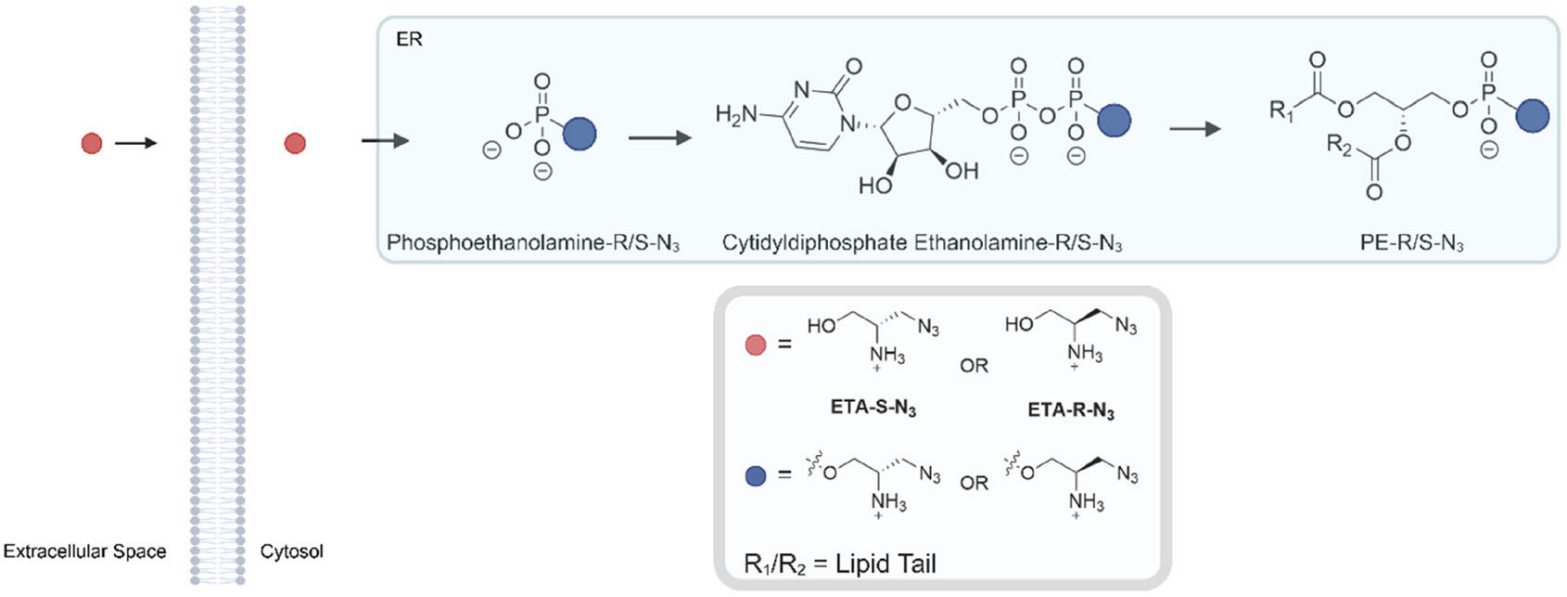
Proposed process for PE labeling using probes **ETA‐R‐N**_**3**_ and **ETA‐S‐N**_**3**_. Probes are expected to first be shuttled into the cytosol, where they will then enter the ER and undergo phosphorylation, forming a phosphoethanolamine analog. Subsequent coupling will yield labeled CDP‐ethanolamine, which will finally be converted to clickable PE‐R/S‐N_3_ for product labeling and tracking.

The desired function for a synthetic probe employed for metabolic labeling is to maximize target product labeling by minimally disturbing metabolic pathways, which can be done by utilizing innate cellular pathways for processes such as cell entry and incorporation. As previously mentioned, despite multiple biosynthetic pathways for producing PE, ETA is the common precursor that enters the Kennedy pathway to produce PE lipids, and therefore this is the substrate on which we based our probes. Despite the innate positive charge of ETA at physiological pH, which could pose problems for crossing the plasma membrane, mammalian cells alleviate this issue by utilizing energy‐dependent transporters embedded in the cell membrane [35]. Therefore, it is important to minimize alterations to probe structures to avoid interference with cellular uptake. We elected to perform initial metabolic incorporation studies in the yeast *S. cerevisiae*, which shares similarities in genes, enzymes, and lipid metabolism pathways with mammalian cells [36]. Although studies of *S. cerevisiae*‐specific ETA transport mechanisms are limited, their cells also contain active transporter proteins, indicating that the shuttling process is likely conserved [5]. Our probe design leverages this proposed cellular uptake by closely mimicking the native structure of ETA. Following cell entry, we expected that our synthetic probes, **ETA‐R‐N**_**3**_ and **ETA‐S‐N**_**3**_, would infiltrate lipid metabolism via the Kennedy pathway by first undergoing phosphorylation catalyzed by ETA kinase to produce a labeled version of ETA phosphate (PEtN), then coupling with cytidine triphosphate (CTP) to form CDP‐ETA, and finally being converted to labeled PE (Figure 1) [37, 38].

To enable post‐derivatization of PE products, probes **ETA‐R‐N**_**3**_ and **ETA‐S‐N**_**3**_ both contain an added azidomethylene group similar to previously successful modifications. Of the two potential carbons on ETA to install the azidomethylene, we speculated that maximizing distance from the primary alcohol could aid probe infiltration by reducing potential steric interactions, as the alcohol moiety undergoes phosphorylation during lipid formation. Furthermore, we recognized that the addition of the azide tag at this position introduces a new stereocenter in these probes. To address this potential issue, we devised a synthetic route to produce both stereoisomers independently and determine if the newly added stereocenter affects labeling within chiral enzyme environments.

**SCHEME 1:**
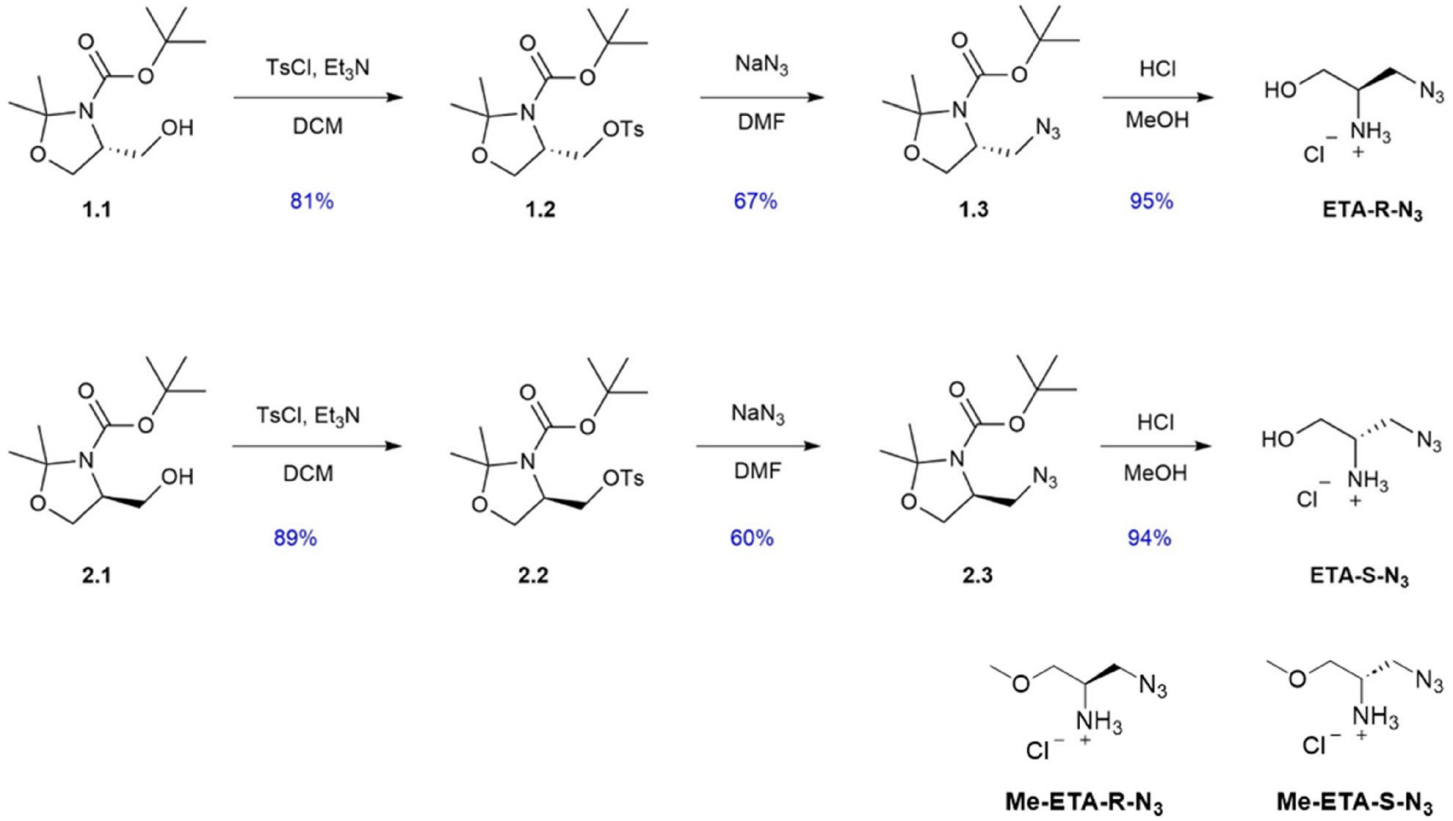
Synthetic route to **ETA‐R‐N**_**3**_ and **ETA‐S‐N**_**3**_ probes. These compounds were obtained by tosylation of **1.1**/**2.1** to **1.2**/**2.2**, azide substitution to **1.3**/**2.3**, and acidic deprotection **ETA‐R‐N**_**3**_/**ETA‐S‐N**_**3**_. Compounds **Me‐ETA‐R‐N**_**3**_ and **Me‐ETA‐S‐N**_**3**_ were also synthesized as negative controls (SI pages S8–S10).

The synthetic route to enantiomeric probes **ETA‐R‐N**_**3**_ and **ETA‐S‐N**_**3**_ is shown in Scheme 1. Both enantiomers of *tert*‐butyl (R/S)‐4‐(hydroxymethyl)‐2,2‐dimethyloxazolidine‐3‐carboxylate starting materials (**1.1** and **2.1**, respectively) were chosen because these enable selective modifications that can be followed by complete acidic deprotection after azide substitution. Both starting materials initially underwent tosylation of the primary alcohol to produce **1.2**/**2.2**, followed by subsequent azide substitution resulting in **1.3/2.3**. Acidic deprotection culminated in the formation of the desired probes **ETA‐R‐N**_**3**_ and **ETA‐S‐N**_**3**_. We further utilized these final structures to synthesize negative control compounds, **Me**‐**ETA‐R‐N**_**3**_ and **Me‐ETA‐S‐N**_**3**_, in which the hydroxyl group is methylated, blocking the phosphorylation site required to biosynthesize PE (SI pages S8–S10).

### 2.1 Probe Effects on Cell Growth

**FIGURE 2:**
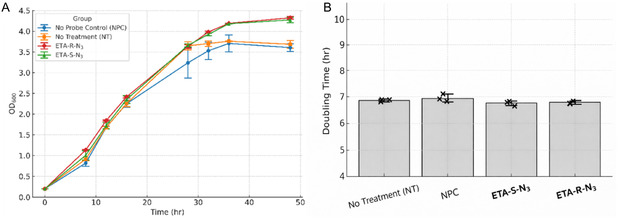
Cell growth results with and without probe treatment. (A) No treatment (NT), no probe control (NPC), **ETA‐S‐N_3_**, and **ETA‐R‐N_3_** groups were incubated with cells beginning at an OD_600_ of 0.2 and growth was measured over a period of 48 h. Probes were incubated so the final concentration was 1 mM. The results showed minimal variation in cell growth upon probe treatment. Holm‐corrected Welch ANOVA values identified significant group differences at 36 h ($p=3.09\times 10^{-5}$) and 48 h (p=0.0380). Games–Howell post hoc testing showed that **ETA‐S‐N**_**3**_ and **ETA‐R‐N**_**3**_ were higher than NT at 36 h ($p=7.20\times 10^{-6}$ and $p=9.33\times 10^{-6}$, respectively). At 48 h, **ETA‐S‐N**_**3**_ and **ETA‐R‐N**_**3**_ were higher than NT (p=0.0304 and p=0.0385) and NPC (p=0.0194 and p=0.0283), respectively. (B) Doubling times (0–18 h) calculated for the same four groups. Error bars represent mean ± standard deviation, with individual triplicate values each shown with an x. Statistical analysis was performed using Welch ANOVA with Games–Howell post‐hoc test. All pairwise comparisons were not significant (ns).

Following the synthesis of **ETA‐R‐N**_**3**_ and **ETA‐S‐N**_**3**_, these probes were incubated with *Saccharomyces cerevisiae* yeast cells (TRY 181 (wild‐type, uraΔhisΔ)) to scrutinize their effects on cell proliferation and overall toxicity via monitoring their growth over an extended period. In this study, four groups of cells were interrogated over the span of 48 h using 2% galactose liquid media. Of the four groups, the first cells underwent no treatment (NT), the second was a no probe control (NPC), which was treated with water (the probe solvent), and the final two consisted of cells treated with probe **ETA‐R‐N**_**3**_ or **ETA‐S‐N**_**3**_ such that the final concentration of probe was 1 mM in media. At the beginning of the 48 h period, all groups shared the same OD_600_ (optical density at 600 nm) of 0.2. Following initial incubation, OD_600_ values were gathered at seven different times during the 48 h period and plotted as a function of time. As shown in Figure 2A, there was no significant shift in growth for any of the groups until the stationary phase; all samples grew in accordance with the NT group during the lag and log phases and exhibited minimal variation in doubling time, which was calculated from 0–18 h (Figure 2B). The negative controls **Me**‐**ETA‐S‐N**_**3**_ and **Me‐ETA‐R‐N**_**3**_ were also evaluated in a similar manner, where the only statistically significant difference in doubling time was between **ETA‐R‐N**_**3**_ and **Me‐ETA‐S‐N**_**3**_ (Figure S7). These results provided evidence that the addition of probes **ETA‐R‐N**_**3**_ and **ETA‐S‐N**_**3**_ at the given concentration did not result in metabolic changes that resulted in toxicity.

### 2.2 Microscopy Imaging Experiments

**FIGURE 3:**
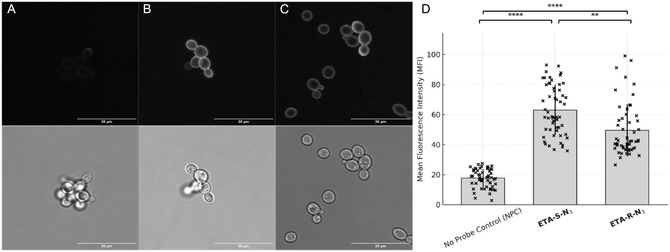
Fluorescence and bright field microscopy images of *S. cerevisiae* cells incubated with NPC (A), **ETA‐S‐N**_**3**_ (B), or **ETA‐R‐N**_**3**_ (C). Cells were fixed, treated with 1 µM **Cy3‐DBCO** for SPAAC coupling, and washed to remove unreacted dye. Images show significant labeling only when probes were added. Images are Z‐stacks containing 12 layered images with fluorescence maximized. MFI values were calculated using at least 8 images with a total of at least 55 cells. Scale bars depict 20 μm (D). Error bars represent mean ± standard deviation, with individual values shown as dots. Post‐hoc Welch's t‐tests with Bonferroni correction showed that both **ETA‐S‐N_3_** and **ETA‐R‐N_3_** groups had significantly higher fluorescence than **NPC** (****p < 0.0001 for each). Additionally, the difference in fluorescence between **ETA‐S‐N_3_** and **ETA‐R‐N_3_** was statistically significant (**p < 0.01;  ****p < 0.0001).

Next, our azido‐ETA probes were incubated with *S. cerevisiae* yeast cells to evaluate their efficacy for labeling cellular membranes and enabling detection using fluorescence microscopy. In addition to providing insight into localization and labeling efficacy, microscopy is also an essential tool for identifying the ideal conditions for cellular incubation of probes for future studies. During experiments in which we varied the duration and concentration of probe treatment, we found that incubation with 1 mM probe over a 2 h period yielded effective labeling (Figures 3 and S1). Longer times including 8 and 12 h incubations were also tested, but yielded minimal fluorescence over NPC samples, suggesting that rapid turnover of phospholipids could lead to cleavage of the clickable headgroup (data not shown). In each case, following probe incubation, cells were washed, fixed with paraformaldehyde, and then treated with dibenzocyclooctyne‐cyanine 3 (**DBCO‐Cy3**), a water‐soluble, orange‐emitting fluorophore containing a strained alkyne for SPAAC labeling of azide‐tagged products. Unreacted dye was then removed using washing steps and cell samples were visualized using a confocal fluorescence microscope. For consistency of metabolic incorporation that could vary depending on the cell culture growth phase, probes **ETA‐S‐N**_**3**_ and **ETA‐R‐N**_**3**_, as well as negative controls **Me**‐**ETA‐S‐N**_**3**_ and **Me‐ETA‐R‐N**_**3**_ were incubated for 2 h for all subsequent microscopy, MS, and TLC studies.

In addition to the clear difference in fluorescence resulting from probe treatment (Figure 3B,C compared to Figure 3A), labeling was also quantified by contrasting the mean fluorescence intensity (MFI) values between NPC studies and the two probes. The NPC cells were shown to exhibit an MFI of 17.7 ± 6.1, comprised mostly of background signal, whereas **ETA‐S‐N**_**3**_ and **ETA‐R‐N**_**3**_ MFIs were 62.9 ± 16.6 and 49.5 ± 17.3, respectively (Figure 3D). These differences in values compared to untreated controls provide evidence that these synthetic probes are effective at entering biological pathways as predicted. Additionally, cells treated with methylated compounds **Me**‐**ETA‐R‐N**_**3**_ and **Me‐ETA‐S‐N**_**3**_ were also imaged along with quantitation of fluorescence labeling. These compounds act as negative controls due to the blocking of the hydroxyl group required for connection to PE. Therefore, they can additionally inform about the extent to which residual probe is retained within cells, providing information about non‐specific fluorescence. The MFI values for these compounds were 25.57 ± 4.63 and 26.17 ± 4.50, respectively. This modest increase in MFI of the methylated compounds compared to the NPC indicates that a marginal amount of clickable probe is persisting following washing steps. More importantly, the MFI values for these compounds are significantly lower than those resulting from probes **ETA‐S‐N**_**3**_ and **ETA‐R‐N**_**3**_, as expected, which provides further validation that those probes are effective at labeling PE lipids. It should also be noted that the majority of fluorescence is located at the plasma membranes of cells. While this supports lipid labeling, it is possible that challenges associated with dye permeability may be obstructing the labeling of PE lipids inside organelles.

### 2.3 Validation of PE Lipid Labeling

**FIGURE 4:**
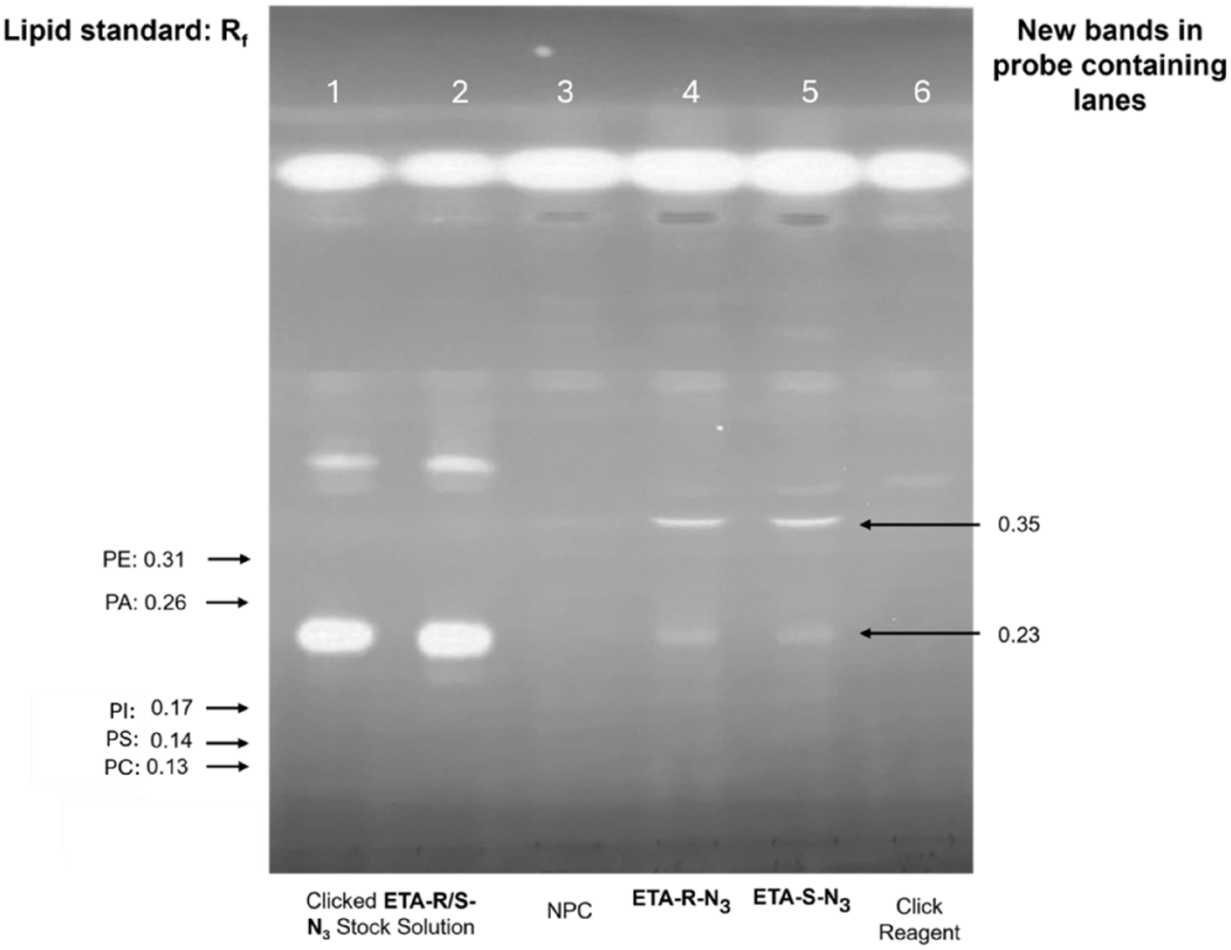
TLC image providing evidence that probes **ETA‐S‐N**_**3**_ and **ETA‐R‐N**_**3**_ label PE in cells. After cellular labeling, lipids were extracted, reacted through CuAAC with **Naphth,** and developed by TLC. Lanes 1 and 2 contain 0.5 mM probe only stock solutions that were clicked with **Naphth**. Lane 3 shows NPC lipid extracts treated with **Naphth**, while lanes 4 and 5 are extracts from cells treated with **ETA‐R‐N**_**3**_ and **ETA‐S‐N**_**3**_ and clicked with **Naphth**, respectively. Lane 6 contained only CuAAC. A new fluorescent band appeared in the **ETA‐R‐N**_**3**_ and **ETA‐S‐N**_**3**_ lanes coinciding with the commercially available lipid standard for PE, which is marked along other commercially available lipids that were spotted on the plate (Figure S4), indicating the formation of a new clicked PE species.

While microscopy provides crucial insight that probe infiltration has occurred, further experiments are necessary to characterize the identities of labeled products. This can be achieved through thin‐layer chromatography (TLC) and liquid chromatography/mass spectrometry (LCMS) analysis of lipid extracts, which have been shown to effectively identify phospholipids through differences in polarity and mass, respectively. For TLC studies, following the 2 h incubation time that was determined during microscopy studies, cellular lipids were extracted and subjected to CuAAC with ethynylnaphthalimide (**Naphth**). **Naphth** is a nonpolar fluorescent dye that was chosen for visualization of labeled lipid bands under UV while minimally affecting *R*_f_ values. We used CuAAC in this case because TLC studies are performed using lipid extracts, rather than intact cells, and therefore copper delivery/toxicity is not an issue. The amount of time allowed for the click reaction and the identity of the TLC mobile phase were both analyzed during experiments. We determined a 3 h CuAAC time to be ideal and that two separate solvent mixtures in tandem could be used for the development of the plate. Plates were first developed with a polar eluant (chloroform/methanol/water/acetic acid = 65:25:4:1, v/v/v/v), which was subsequently switched to a nonpolar eluant mixture (cyclohexane/ethyl acetate = 1:1). The resulting plate was visualized under a UV lamp (Figure 4). The appearance of a new band with an *R*_f_ value (0.35) that closely matched the commercial PE standard was observed from the lipid extracts of both enantiomeric probe‐containing lanes (Figure 4, lanes 4 and 5). While this band appeared at slightly higher *R*_f_ than the PE standard (*R*_f_ = 0.31), this can be explained by the clicked lipid products containing the hydrophobic dye **Naphth** appended to the structure for visualization purposes. This band is notably absent from the NPC lane (Figure 4, lane 3), as well as the lanes correlating to the methylated negative control compounds **Me‐ETA‐S‐N**_**3**_ and **Me‐ETA‐R‐N**_**3**_ (Figure S5). Figure 4 also shows the *R*_f_ values of various commercial lipid standards, including natural PE (indicated by arrows to the left of the plate), for comparison. An additional band is present in lanes (1–2, 4–5) at *R*_f_ = 0.23, but this correlates with the clicked **ETA‐R/S‐N**_**3**_ stock solution, suggesting some residual probe is present. Nevertheless, the formation of a new band with similarities in *R*_f_ values to PE provided initial evidence that probes **ETA‐S‐N**_**3**_ and **ETA‐R‐N**_**3**_ are capable of infiltrating lipid metabolic pathways of *S. cerevisiae* to produce functionalized PE derivatives.

While TLC provides initial insight into the species of phospholipids present, in this case PE, LCMS is invaluable for further confirmation and to identify specific structural details of labeled lipids, such as the lipid tail length or unsaturation. For these experiments, following the 2 h incubation period, lipids were extracted from cells, but in this case were subjected to LCMS analysis. Due to the zwitterionic nature of PE, we scanned for and detected PE species in both positive and negative mode as both the [M − H]^−^ or [M + H]^+^ ions. In addition to mass, a similar retention time for the natural and tagged lipids was observed, indicating comparable polarity between the labeled and unlabeled species, which is expected due to their related structures. Through these experiments we detected mass peaks correlating with various labeled PE lipid species (Table S1; representative spectra in Figure S6), indicating that **ETA‐S‐N**_**3**_ and **ETA‐R‐N**_**3**_ are effective at infiltrating cellular biochemical pathways, specifically the Kennedy pathway. In addition, we noticed a complete overlap between labeled phospholipids from each probe, demonstrating that the stereochemical configuration of the stereocenter does not impact lipid labeling properties. Interestingly, we did not observe any labeled PC lipid species either in positive or negative mode during LCMS studies. This was notable because labeled PE molecules could potentially undergo methylation into corresponding PC structures, particularly due to the abundant nature of the latter lipid. On the other hand, we did observe mass peaks correlating with phosphatidyl‐*N*,*N*‐dimethylethanolamine (PDME) species. Therefore, we hypothesize that the addition of an azidomethylene group could sterically hinder complete methylation to generate PC products.

## 3 Conclusions

The culmination of these studies indicates that our synthetic azide‐tagged ETA probes **ETA‐S‐N**_**3**_ and **ETA‐R‐N**_**3**_ are capable of infiltrating cellular pathways and acting as substrate mimetics to produce functionalized labeled PE species. Cellular growth studies indicated biocompatibility and minimal variability in cell proliferation of these probes in yeast cells. Fluorescence microscopy experiments showed that these probes were effective for the labeling of yeast cellular membranes. Furthermore, the ability of these probes to successfully undergo structural modifications to produce labeled PE molecules was supported by both TLC and MS experiments, which provided insight into the various PE species that were labeled. While quantitation of lipid labeling was considered, such experiments are complicated since they often require synthetic versions of each tagged lipid as internal standards, particularly since chromatographic retention times are similar for labeled and natural lipids. While this is a limitation of the current study, the primary focus of this work entailed qualitative validation of lipid labeling. Finally, our study of both stereoisomers of the newly found probes indicates that the stereochemistry at the position bearing the new azidomethylene group does not affect labeling activity. Therefore, it may be suitable and more convenient to employ these probes as a racemic mixture in future studies. These results demonstrate the effectiveness of **ETA‐S‐N**_**3**_ and **ETA‐R‐N**_**3**_ for infiltrating PE lipid metabolism, marking them as promising candidates for further investigations into lipid pathways and other lipid‐related cellular processes.

## Funding

This work was supported by the National Science Foundation (Grant NSF‐CHE‐2310263).

## Conflicts of Interest

The authors declare no conflicts of interest.

## Supporting information

Additional synthetic information such as procedures and characterization data for compounds are described in the Supporting Information.

## Data Availability

The data that support the findings of this study are available from the corresponding author upon reasonable request.
