# Supplementary material for: Metabolic Labeling of Phosphatidylethanolamine Lipids in Cells Using Clickable Ethanolamine Analogs
Source: Chembiochem. 2026 Aug 14;27(15):e70501. doi: 10.1002/cbic.70501 (PMC13475593; doi:10.1002/cbic.70501)
Supplement: Supplementary file 1 [file CBIC-27-e70501-s001.pdf]

Supplemental Information

Metabolic Labeling of Phosphatidylethanolamine Lipids in Cells using Clickable Ethanolamine Analogs

Robert J. Maraski[a], Jinchao Lou[a], Todd B. Reynolds[b], and Michael D. Best[a]*

[a]R. J. Maraski; J. Lou; Prof. M. D. Best; Department of Chemistry; University of Tennessee, Knoxville; 1420 Circle Drive, Knoxville, TN, 37996, USA

E-mail: mdbest@mail.tennessee.edu

[b]Prof. T. B. Reynolds; Department of Microbiology; University of Tennessee, Knoxville; 1311 Cumberland Avenue, Knoxville, TN, 37996, USA

[a] Department of Chemistry, University of Tennessee, Knoxville, TN, 37996, USA

[b] Department of Microbiology, University of Tennessee, Knoxville, TN, 37996, USA

Table of Contents

Pg S2-S4: General Experimental

Pg S5-S10: Synthetic Schemes and Procedures

Pg S11-S18: Supplementary Figures

Pg S19: Supplementary References

Pg S20-S42: Spectra of Synthetic Compounds

General Experimental

Reagents were generally purchased from commercial suppliers, including Sigma-Aldrich, Fisher Scientific, and Ambeed, and were used as received without additional purification. Lipid standards—L-α-phosphatidylcholine (PC, mixed isomers from chicken egg), L-α-phosphatidic acid (PA, sodium salt from chicken egg), L-α-phosphatidylserine (PS, porcine brain, sodium salt), 1,2-dioleoyl-*sn*-glycero-3-phosphatidylethanolamine (DOPE), and 1,2-dipalmitoyl-*sn*-glycero-3-phospho-(1-*myo*-inositol) (16:0 PI, ammonium salt)—were obtained from Avanti Polar Lipids (Alabaster, AL). Cy3-DBCO (dibenzocyclooctyne-cyanine) was purchased from Click Chemistry Tools. 4-Ethynyl-N-ethyl-1,8-naphthalimide was sourced from Fisher Scientific. Silica gel TLC plates (0.25 mm, 20 × 20 cm, non-fluorescent) and 230–400 mesh silica gel for column chromatography were obtained from Sorbent Technologies (Norcross, GA). Anhydrous solvents were supplied through a Pure Solv MD-7 purification system (Innovative Technology, Newburyport, MA). NMR spectra were recorded on Varian 500 MHz instruments, and mass spectrometric data were collected using a JEOL DART-AccuTOF system. Ultrapure water was generated using a Millipore purification system (≥ MW×cm triple purification). Samples under 5 mg were measured using a Mettler Toledo XS105 dual-range analytical balance.

***S. Cerevisiae* Cell Growth:** *S. cerevisiae* TRY181 (wild-type, uraΔhisΔ) cells were plated on YPD agar (1% yeast extract, 2% peptone, 2% dextrose, 2% agar) and incubated at 30 °C. Colonies developed after approximately 24 hours. A single colony was then used to inoculate 5 mL of 2% galactose minimal medium in a glass culture tube, using one to two sterile loops of cells. Cultures were grown at 30 °C with shaking at 225 rpm to ensure adequate aeration for ~24 hours. To monitor sterility, a parallel control tube containing only medium and streaked with a sterile loop was prepared each time. Galactose minimal medium (2%, YNB, galactose as the carbon source) was prepared by dissolving 3.35 g Yeast Nitrogen Base (without amino acids or ammonium sulfate), 10 mg uracil, 10 mg L-histidine, and 10 g galactose in 1000 mL Milli-Q water. After full dissolution, the solution was sterilized through a pre-sterilized Millipore vacuum filtration system and transferred into 50 mL Falcon tubes for use to minimize contamination.

Treatment ETA-R/S-N_3 Probes in *S. cerevisiae*: Stock solutions of the **ETA-R/S-N_3** and **Me-ETA-R/S-N_3** probes were prepared in water at a concentration of 50 mM and stored at 4 °C. Overnight yeast cultures grown in 2% galactose medium were diluted into fresh medium to an OD_{600} of 0.2, and the probes were added to reach a final concentration of 1 mM in either 5 mL (for microscopy experiments) or 10 mL (for TLC or MS analyses). For negative controls, an equivalent volume of water was added instead of the probe. Cultures were incubated for 2 hours. To maintain sufficient aeration, culture volume never exceeded one-fifth of the tube capacity, and cells were shaken at 30 °C and 225 rpm.

Fluorescence Imaging of Labeled Lipids in *S. cerevisiae*: After incubation with the probe, cultures were transferred into 15 mL conical tubes and centrifuged at 3000 × g for 5 minutes at 4 °C. Pellets were washed with 1 mL of 1× PBS, then resuspended and fixed in 1 mL of 3.7% paraformaldehyde (PFA) in PBS for 15 minutes at room temperature. Following fixation, cells were washed once with 1 mL of 1× PBS and moved

into a clean 15 mL conical tube containing 0.5 mL of 1× PBS. Cy3-DBCO (0.4 mM stock in Milli-Q water) was added to achieve a final concentration of 1 μM, and samples were incubated for 1 hour at 30 °C with shaking at 225 rpm.

Cells were washed twice with 0.5 mL 1× PBS, vortexed, and centrifuged at 3000 × g. Final resuspension in 0.2 mL 1× PBS (pH 7.4) yielded the click-labeled yeast samples. For microscopy, 3 μL of each sample was placed on a slide, covered with a coverslip, sealed with acrylic nail polish, and imaged using a Leica SP8 White Light Laser confocal microscope equipped with a 63× oil objective. Differential interference contrast (DIC) imaging was used to identify cell locations, and samples were visualized at a zoom factor of 1 or 4. Fluorescence measurements were obtained using 554 nm excitation and collected with a HyD detector between 559–620 nm. Laser power, gain, and offset were kept constant across all conditions.

Images of *S. cerevisiae* were acquired as Z-stacks of 7–14 slices at 1.0 μm intervals, followed by Lightning deconvolution (S14) in Leica Application Suite (LAS) v4.4. All images were processed identically using FIJI (v2.14.0/1.54f). Quantification was performed using Z-stack images collected at 4× zoom, analyzing at least 33 cells for the negative control, 67 cells for **ETA-R-N$_3$**, 86 cells for **ETA-S-N$_3$**, 49 cells for **Me-ETA-R-N$_3$**, and 40 cells for **Me-ETA-S-N$_3$** from a minimum of 10 images per condition using ImageJ.

Lipid Extraction for Mass Spectrometry and Fluorescence-based Thin Layer Chromatography: Lipid extraction was carried out using a previously reported protocol with slight modifications.[1] After probe incubation, 10 mL of cell culture were placed on ice, and 0.5 mL of ice-cold 100% (w/v) trichloroacetic acid (TCA) was added. Samples were incubated for 1 hour, then transferred into 15 mL conical tubes and centrifuged to remove media and TCA. Pellets were washed three times with 5 mL Milli-Q water. The resulting cells were either processed immediately for extraction or stored at −20 °C.

For extraction, cell pellets at room temperature were resuspended in 0.5 mL of lipid extraction solution composed of 100% ethanol, water, diethyl ether, pyridine, and 4.2 N NH_4OH (15:15:5:1:0.018, v/v). Suspensions were transferred to 2 mL microcentrifuge tubes, vortexed for 30 seconds, and incubated at 55 °C for 15 minutes. Tubes were briefly opened midway through heating to release pressure. After incubation, samples were centrifuged, and the supernatants were collected into new tubes. The extraction step was repeated with an additional 0.5 mL of extraction solution, yielding a total extract volume of 1 mL. Final extracts were evaporated under a stream of nitrogen.

Ultra-High Performance Liquid Chromatography High Resolution Mass Spectrometry (UHPLC-HRMS) and Analysis: Dried lipid extracts obtained from the extraction protocol were reconstituted in 100 μL of 73:23:3 $CHCl_3$:MeOH:H_2O and transferred to GC vials for analysis.[2] Lipid profiling was carried out using a Vanquish UHPLC system (Dionex, Sunnyvale, CA) coupled to an Exploris 120 high-resolution mass spectrometer (Thermo Scientific), operated in both positive and negative ion modes, following a previously reported method with modifications. Separation was achieved on a Thermo C30 column (2.1 × 150 mm, 2.6 μm) with a 4 μL injection volume. The chromatographic gradient was as follows: 0–2 min, 30% B; 3 min,

40% B; 7 min, 50% B; 8 min, 60% B; 10 min, 65% B; 12 min, 68% B; 13 min, 70% B; 16 min, 75% B; 20 min, 80% B; 23–28 min, 98% B; 32 min, 100% B; 34 min, 90% B; and 35–40 min, 30% B, all at a flow rate of 0.15 µL/min. Solvent A consisted of 60:40 water:acetonitrile with 10 mM ammonium formate and 0.1% formic acid. Solvent B consisted of 90:10 isopropanol:acetonitrile containing 10 mM ammonium formate and 0.1% formic acid. The mass spectrometer was run in data-dependent acquisition (DDA) mode. Full scans from m/z 150–2000 were collected at 120,000 resolution (200 m/z), while MS^2 spectra were obtained at 60,000 resolution with stepped collision energies of 20, 50, and 75 eV. Samples were introduced via a heated electrospray ionization source with static spray voltages of 3500 V (positive mode) and 2500 V (negative mode). Additional HESI source parameters included: sheath gas 35 Arb, auxiliary gas 7 Arb, and ion transfer tube temperature 320 °C. Mass calibration was performed before sample acquisition in both ionization modes using the commercially available Pierce™ FlexMix™ calibration solution (Thermo Scientific) to maintain high mass accuracy throughout data collection.[3] Raw data files were converted to mzML format using MSConvert, and full-scan data were analyzed with El-MAVEN. For peak assignment, retention times of labeled lipids were compared to those of their corresponding native species. Labeled species were identified from a curated compound list, using the C^{12} isotopologue as the parent ion and an accuracy tolerance of ±5 ppm.

CuAAC for Fluorescence-based Thin Layer Chromatography: Dried lipid extracts obtained from the previous extraction step were resuspended in 16 µL of $CHCl_3$. To prepare an 8.60 mM Cu(I) click reagent, 3.80 mg of $Cu(I)(MeCN)_4BF_4$ was dissolved in 605 µL acetonitrile and 800 µL ethanol. A portion of this solution (200 µL) was mixed with 10 µL of **Naphth** solution (22.5 µM in $CHCl_3$/DMSO), vortexed for 30 seconds, and used as the click mixture. For CuAAC labeling, 20 µL of click mixture was added to each lipid extract (in 16 µL $CHCl_3$), as well as to aliquots of unreacted **ETA-R/S-N_3** and **Me-ETA-R/S-N_3** probe stocks (1 mM). Samples were flushed with N_2, vortexed, centrifuged, and incubated in a 42 °C water bath for 3 hours.

Following CuAAC, samples were vortexed and centrifuged (4,600 × g). The resulting clicked mixtures were applied to thin-layer chromatography (TLC) plates in 5 µL increments with drying between applications (10 µL total for lanes containing probe only; full sample volume for NPC or probe-treated cells). Lipids were separated on 20 × 20 cm glass TLC plates coated with 0.25 mm silica gel lacking fluorescent indicator. Phospholipid standards (PS, PC, PA, PI, DOPE; 3–5 mM in $CHCl_3$/MeOH) were spotted on the same plate in 4–10 µL aliquots.

Plates were air-dried and developed in chloroform/methanol/water/acetic acid (65:25:4:1, v/v/v/v) until reaching an R_f = 0.55, then dried for one hour and further developed in 1:1 cyclohexane/ethyl acetate to a final R_f = 1. After drying, TLC plates were imaged under 365 nm UV illumination. Images were recorded with an iPhone camera and processed in ImageJ. Grayscale images were generated by splitting RGB channels and selecting the red channel. Clicked lipid spots were marked with a pencil prior to primulin

visualization. Plates were then dipped in primulin stain (5 mg primulin per 100 mL of 9:1 acetone/water) and re-imaged.

Synthetic Procedures and Characterization Data

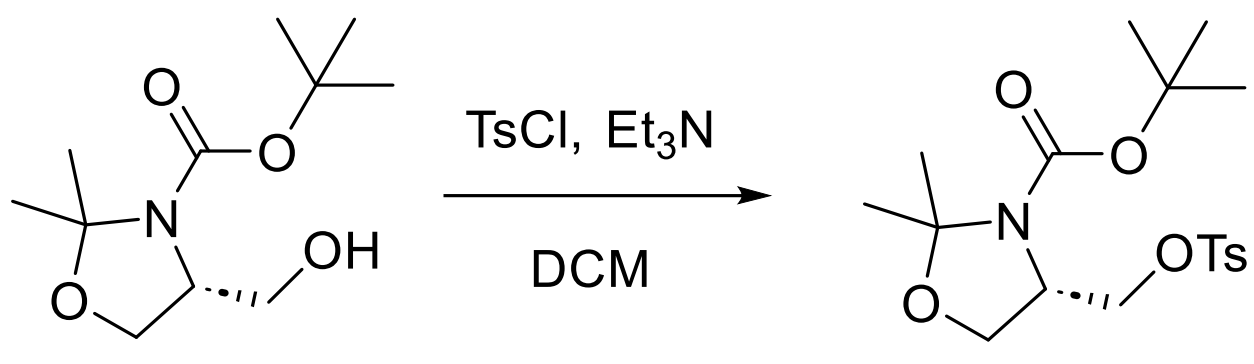

1.1 **1.2**

(S)-*tert*-Butyl 2,2-dimethyl-4-((tosyloxy)methyl)oxazolidine-3-carboxylate (1.2)

tert-Butyl (R)-4-(hydroxymethyl)-2,2-dimethyloxazolidine-3-carboxylate (0.2 g, 0.865 mmol) was dissolved in dry dichloromethane (DCM) at RT in a 100 mL round-bottomed flask (RBF) under Ar. *p*-Toluenesulfonyl chloride (TsCl, 0.248 g, 1.3 mmol) and triethylamine (TEA, 0.132 g, 0.18 mL, 1.3 mmol) were added, and the mixture was stirred overnight. The crude was concentrated, and then subjected to column chromatography and a gradient elution from hexanes to 25% ethyl acetate (EtOAc) in hexanes was used to yield **1.2** as a yellow oil (0.2688 g, 0.697 mmol, 81% yield). R_f = 0.45 (25% EtOAc in hexanes).

^{1}H NMR 500 MHz ($CDCl_3$) δ 7.79 (d, J = 8.2 Hz, 2H), 7.33 (t, *J* = 8.7 Hz, 2H), 4.20-3.70 (m, 5H), 2.45 (s, 3H), 1.48 (dd, *J* = 20.6, 6.8 Hz 6H), 1.42 (d, *J* = 26.7 Hz 9H). ^{13}C NMR (126 MHz, $CDCl_3$) δ 151.26, 145.12, 132.67, 127.89, 94.16, 80.48, 67.84, 64.90, 55.49, 28.26, 27.28, 26.67, 24.19, 22.87, 21.62. HRMS-DART: $[M+H]^+$ calcd for $C_{18}H_{27}NO_6S$: 386.1593; found: 386.1507.

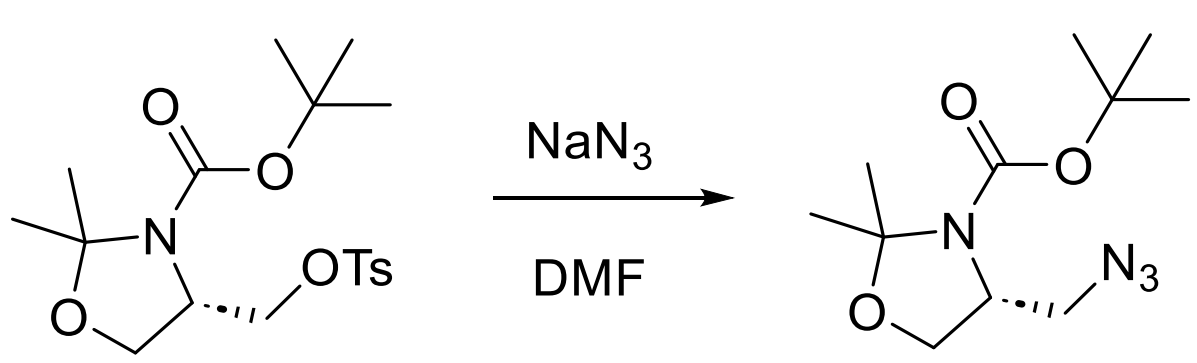

1.2 **1.3**

(R)-*tert*-Butyl 4-(azidomethyl)-2,2-dimethyloxazolidine-3-carboxylate (1.3)

Compound **1.2** (0.8 g, 2.1 mmol) and sodium azide (0.4 g, 5 mmol) were weighed into a 100 mL RBF under argon and then dissolved with 20 mL dry DMF. The reaction was stirred overnight at 80℃ before being cooled to room temperature. The solid formed in the RBF was filtered off through a celite pad and the filtrated was concentrated under vacuum. The resulting crude was subjected to column chromatography packed with SiO_2 with a gradient elution of hexanes to 25% EtOAc in hexanes to yield **1.3** as a yellow oil (0.398 g, 1.55 mmol, 74% yield). R_f = 0.55 (25% EtOAc in hexanes).

^{1}H NMR 500 MHz ($CDCl_3$) δ 3.96 (dd, *J* = 9.3, 5.9 Hz, 1H), 3.89 (dd, *J* = 9.1, 1.4 Hz, 1H), 3.52 (dd, *J* = 28.9, 10.1 Hz, 1H) 3.31 (dt, J = 74.1, 10.1 Hz, 1H) 1.61-1.51 (d, *J* = 17.7 Hz, 6H), 1.47 (s, 9H). ^{13}C NMR

(126 MHz, $CDCl_3$) δ 152.00, 93.68, 80.43, 65.09, 56.37, 50.97, 28.21, 27.03, 26.45, 24.24, 22.82. HRMS-DART: $[M+H]^+$ calcd for $C_{11}H_{20}N_4O_3$: 257.1569; found: 257.1565.

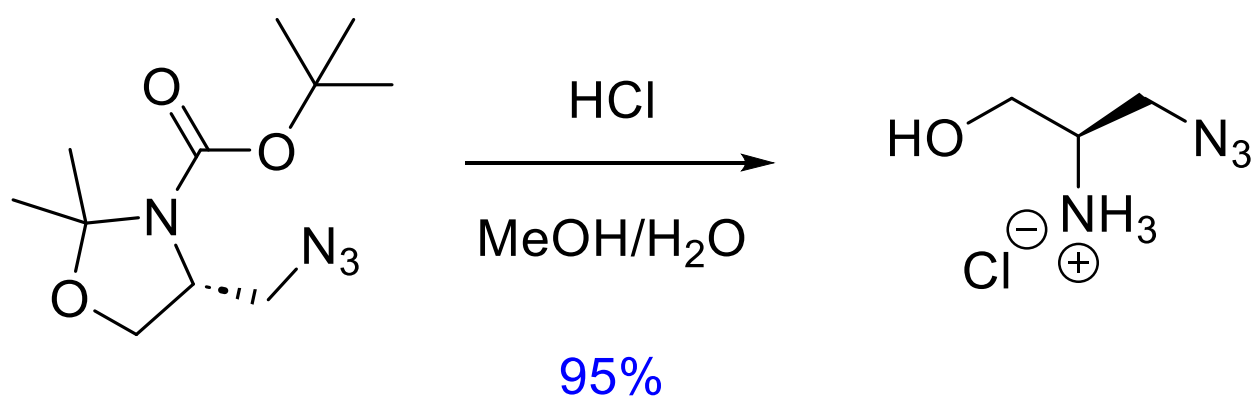

1.3 **ETA-R-N3**

(R)-1-Azido-3-hydroxypropan-2-aminium chloride (ETA-R-N$_3$)

Compound **1.3** (107.8 mg, 0.421 mmol) was added to a 100 mL RBF and dissolved with 10 mL MeOH under Ar. 8M HCl (3.33 mL of 12M HCl dissolved in 1.67 mL H_2O) was added to the solution dropwise and allowed to stir overnight. The reaction was cooled to 0℃ and diluted with 6 mL MeOH and then concentrated under vacuum. The remaining crude was subjected to reverse phase chromatography with an elution of 100% water to yield a yellow film (46 mg, 0.40 mmol, 95% yield). R_f = 0.9 (1:1 MeOH:H_2O) imaged with Ninhydrin stain.

^{1}H NMR (500 MHz, CD_3OD) δ 3.74 (dd, J = 2.2 Hz, 2H), 3.64 (t, *J* = 4.2 Hz, 2H), 3.35 (q, *J* = 5.5 Hz, 1H). ^{13}C NMR (126 MHz, $CDCl_3$) δ 58.96, 52.15, 49.47. HRMS-DART: $[M+H]^+$ calcd for $C_3H_8N_4O$: 117.0732; found: 117.0755.

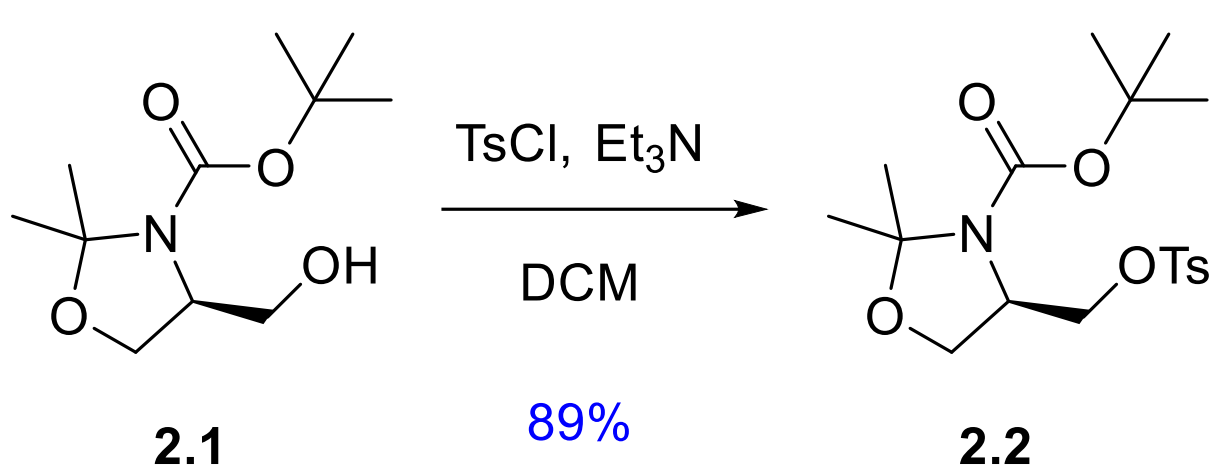

2.1 **2.2**

(R)-*tert*-Butyl 2,2-dimethyl-4-((tosyloxy)methyl)oxazolidine-3-carboxylate (2.2)

(S)-*tert*-Butyl 4-(hydroxymethyl)-2,2-dimethyloxazolidine-3-carboxylate (**2.1**, 0.5 g, 2.16 mmol) was dissolved with dry DCM at room temperature in a 100 mL RBF under Ar. TsCl (0.618 g, 3.24 mmol) and TEA (0.328 g, 0.45 mL, 3.24 mmol) were added, and the mixture was stirred overnight. The crude was concentrated, and then subjected to column chromatography, and a gradient elution from hexanes to 25% EtOAc in hexanes was used to yield **2.2** as a yellow oil (0.7445 g, 1.92 mmol, 89% yield). R_f = 0.45 (25% EtOAc in hexanes).

^{1}H NMR 500 MHz ($CDCl_3$) δ 7.79 (d, J = 8.2 Hz, 2H), 7.33 (t, *J* = 8.7 Hz, 2H), 4.20-3.70 (m, 5H), 2.45 (s, 3H), 1.48 (dd, *J* = 20.6, 6.8 Hz 6H), 1.42 (d, *J* = 26.7 Hz 9H). HRMS-DART: $[M+H]^+$ calcd for $C_{18}H_{27}NO_6S$: 386.1593; found: 386.1583.

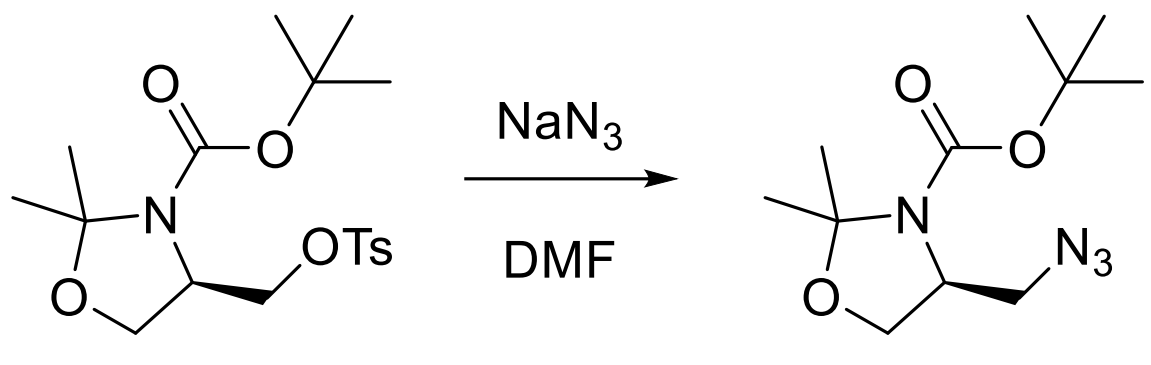

2.2 67% **2.3**

(S)-*tert*-Butyl 4-(azidomethyl)-2,2-dimethyloxazolidine-3-carboxylate (2.3)

Compound **2.2** (0.6623 g, 1.71 mmol) and sodium azide (0.556 g, 8.55 mmol) were weighed into a 100 mL RBF under argon and then dissolved with 10 mL dry DMF. The reaction was stirred overnight under reflux before being cooled to room temperature. The solid formed in the RBF was filtered off through a celite pad, and the filtrate was concentrated under vacuum. The resulting crude was subjected to column chromatography packed with SiO_2 with a gradient elution of hexanes to 25% EtOAc in hexanes to yield **2.3** as a yellow oil (0.161 g, 0.64 mmol, 67% yield). R_f = 0.55 (25% EtOAc in hexanes).

^{1}H NMR 500 MHz ($CDCl_3$) δ 3.96 (dd, *J* = 9.3, 5.9 Hz, 1H), 3.89 (dd, *J* = 9.1, 1.4 Hz, 1H), 3.52 (dd, *J* = 28.9, 10.1 Hz, 1H) 3.31 (dt, J = 74.1, 10.1 Hz, 1H) 1.61-1.51 (d, *J* = 17.7 Hz, 6H), 1.47 (s, 9H). ^{13}C NMR (126 MHz, $CDCl_3$) δ 152.14, 94.27, 80.68, 65.62, 56.46, 52.25, 51.09, .28.37, 27.16, 26.61, 24.37, 22.97. HRMS-DART: $[M+H]^+$ calcd for $C_{11}H_{20}N_4O_3$: 257.1569; found 257.1572.

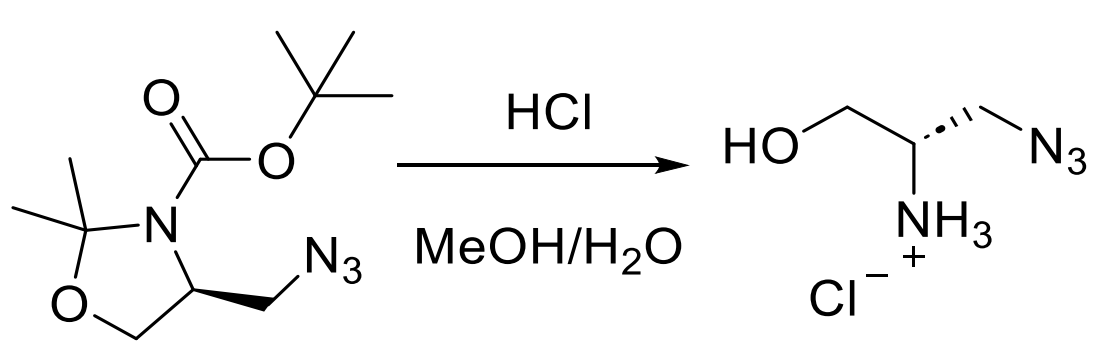

2.3 94% **ETA-S-N3**

(S)-1-Azido-3-hydroxypropan-2-aminium chloride (ETA-S-N_3)

Compound **2.3** (0.161 g mg, 0.64 mmol) was added to a 100 mL RBF and dissolved with 10 mL MeOH under Ar. 8M HCl (3.33 mL of 12M HCl dissolved in 1.67 mL H_2O) was added to the solution dropwise and allowed to stir overnight. The reaction was cooled to 0℃ and diluted with 6 mL MeOH, and then concentrated under vacuum. The remaining crude was subjected to reverse-phase chromatography with an elution of 100% water to yield a yellow film (69 mg, 0.59 mmol, 94% yield). R_f = 0.9 (1:1 MeOH:H_2O) imaged with Ninhydrin stain.

^{1}H NMR (500 MHz, CD_3OD) δ 3.74 (dd, J = 2.2 Hz, 2H), 3.64 (t, J = 4.2 Hz, 2H), 3.35 (q, J = 5.5 Hz, 1H). ^{13}C NMR (126 MHz, $CDCl_3$) δ 58.91, 52.13, 49.40. HRMS-DART: $[M+H]^+$ calcd for $C_3H_8N_4O$: 117.0732; found: 117.07288.

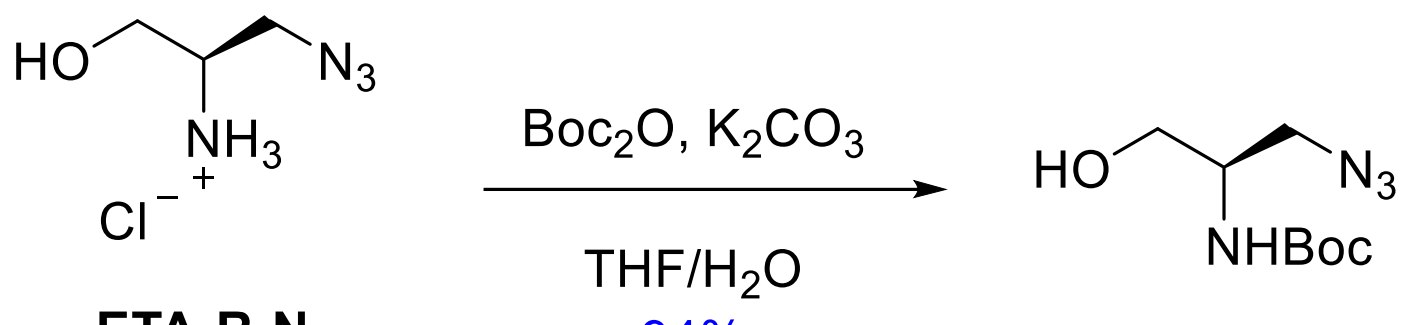

(R)-*tert*-Butyl (1-azido-3-hydroxypropan-2-yl)carbamate (1.4)

ETA-R-N$_3$ (0.4112 g, 2.77 mmol, 1 equiv.) was added to a 100 mL round bottom flask under argon protection and dissolved in water (5 mL) and THF (5 mL). The mixture was cooled to 0 ℃, and Boc_2O (0.663 g, 3.04 mmol, 1.1 equiv.) and K_2CO_3 (1.15 g, 8.3 mmol, 3 equiv.) were added, after which the reaction was allowed to stir for 4 hours at room temperature. The crude was transferred to a separatory funnel, and extracted 3x with DCM, washed with brine, and dried over sodium sulfate. This mixture was concentrated and subjected to flash chromatography with a gradient up to 50% EtOAc/hexanes to yield the colorless oil **1.4** (0.5944 g, 2.60 mmol, 94% yield)

^{1}H NMR 500 MHz ($CDCl_3$) δ 5.29 (s, 1H), 3.77-3.51 (m, 4H), 3.48-3.31 (m, 2H), 1.38 (s, 9H). ^{13}C NMR (126 MHz, $CDCl_3$) δ 155.90, 80.05, 61.88, 51.33, 28.27.

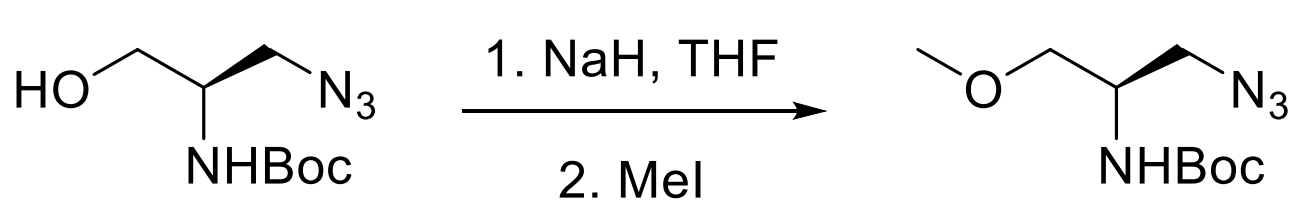

(R)-*tert*-Butyl (1-azido-3-methoxypropan-2-yl)carbamate (1.5)

Compound **1.4** (0.1569 g, 0.726 mmol, 1 equiv.) was added to a 100 mL round bottom flask under argon protection and dissolved in THF (6 mL). The solution was cooled to 0 ℃, and NaH (60% dispersion in paraffin oil, 35 mg, 0.87 mmol, 1.2 equiv.) was added and allowed to stir for 30 minutes. MeI (52 μL. 0.834 mmol, 1.15 equiv.) was added dropwise, and the mixture was allowed to warm to room temperature and stir for 2 hours. The solution was quenched with saturated NH_4Cl, extracted 3X with DCM, washed with brine, and dried over sodium sulfate. This was concentrated under vacuum and subjected to flash chromatography with a gradient up to 30% EtOAc in hexanes to yield the colorless oil **1.5** (0.1016 g, 0.443 mmol, 61% yield).

^{1}H NMR 500 MHz ($CDCl_3$) δ 4.95 (s, 1H), 3.82 (s, 1H), 3.46–3.38 (m, 2H), 3.36-3.31 (m, 2H), 3.30 (s, 3H) 1.39 (s, 9H). ^{13}C NMR (126 MHz, $CDCl_3$) δ 155.18, 79.67, 71.32, 58.92, 51.40, 49.65, 28.07.

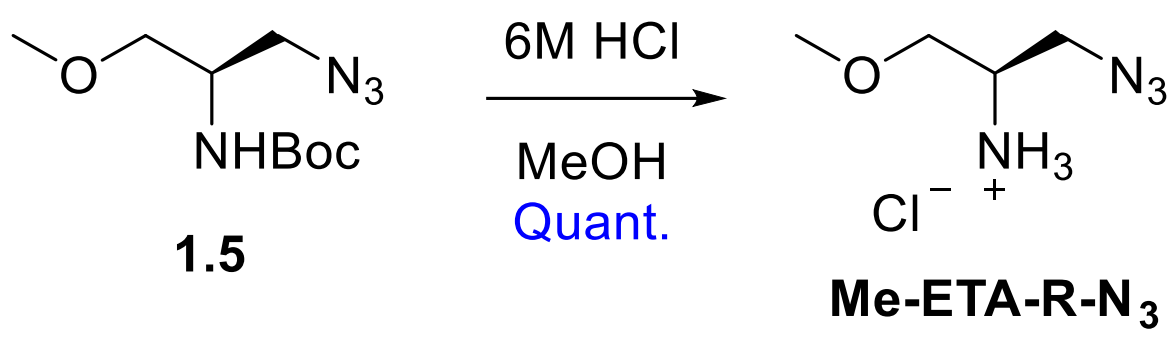

(R)-1-Azido-3-methoxypropan-2-amine hydrochloride (Me-ETA-R-N_3)

Compound **1.5** (0.1016 g, 0.441 mmol) was added to a 100 mL round bottom flask under argon protection and cooled to 0℃. A precooled solution of 6M HCl (8 mL) was added slowly to the flask and stirred for 30 minutes at room temperature. The mixture was diluted with MeOH (10 mL) and concentrated under vacuum to yield **Me-ETA-R-N_3** in quantitative yield as a colorless solid (72.5 mg, 0.44 mmol).

^{1}H NMR 500 MHz (CD_3OD) δ 3.81-3.75 (dd, *J* = 5.6, 1H), 3.71-3.61 (m, 2H), 3.64-3.60 (q, *J* = 6.1, 1H), 3.51 (s, 1H), 3.41 (s, 3H). ^{13}C NMR (126 MHz, CD_3OD) δ 69.21, 58.25, 50.42, 49.50.

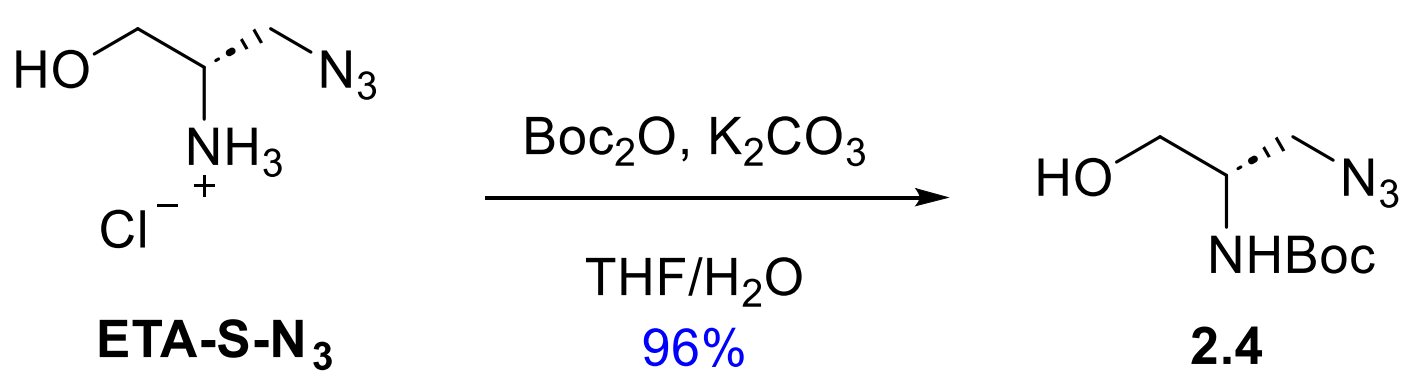

(S)-*tert*-Butyl (1-azido-3-hydroxypropan-2-yl)carbamate (2.4)

ETA-S-N_3 (0.1421 g, 0.931 mmol, 1 equiv.) was added to a 100 mL round bottom flask under argon protection and dissolved in water (4 mL) and THF (4 mL). The mixture was cooled to 0 ℃, and Boc_2O (0.223 g, 1.024 mmol, 1.1 equiv.) and K_2CO_3 (0.283 g, 2.05 mmol, 2 equiv.) were added, after which the reaction was allowed to stir for 4 hours at room temperature. The crude was transferred to a separatory funnel, and extracted 3x with DCM, washed with brine, and dried over sodium sulfate. This mixture was concentrated and subjected to flash chromatography with a gradient up to 50% EtOAc/hexanes to yield the colorless oil **2.4** (0.2555 g, 0.893 mmol, 96% yield)

^{1}H NMR 500 MHz ($CDCl_3$) δ 5.29 (s, 1H), 3.77-3.51 (m, 3H), 3.48-3.31 (m, 2H), 3.19 (bs, 1H), 1.38 (s, 9H). ^{13}C NMR (126 MHz, $CDCl_3$) δ 155.84, 80.13, 61.12, 51.45, 28.44.

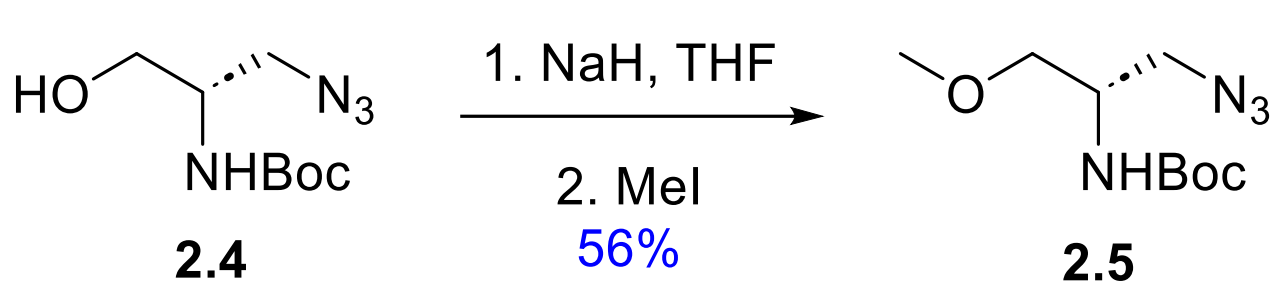

(S)-*tert*-Butyl (1-azido-3-methoxypropan-2-yl)carbamate (2.5)

Compound **2.4** (0.5944 g, 2.75 mmol, 1 equiv.) was added to a 100 mL round bottom flask under argon protection and dissolved in THF (10 mL). The solution was cooled to 0 ℃, NaH (60% dispersion in paraffin oil, 0.132 g, 3.3 mmol, 1.2 equiv.) was added and allowed to stir for 30 minutes. MeI (0.197 mL. 3.16 mmol, 1.15 equiv.) was added dropwise, and the mixture was allowed to warm to room temperature and stir for 2 hours. The solution was quenched with saturated NH_4Cl, extracted 3X with DCM, washed with brine, and dried over sodium sulfate. This was concentrated under vacuum and subjected to flash chromatography with a gradient up to 30% EtOAc in hexanes to yield the colorless oil **2.5** (0.3575 g, 1.54 mmol, 56% yield).

^{1}H NMR 500 MHz ($CDCl_3$) δ 4.95 (s, 1H), 3.82 (s, 1H), 3.46–3.38 (m, 2H), 3.36-3.31 (m, 2H), 3.30 (s, 3H) 1.39 (s, 9H). ^{13}C NMR (126 MHz, $CDCl_3$) δ 155.20, 79.81, 71.33, 59.02, 51.45, 49.65, 28.37.

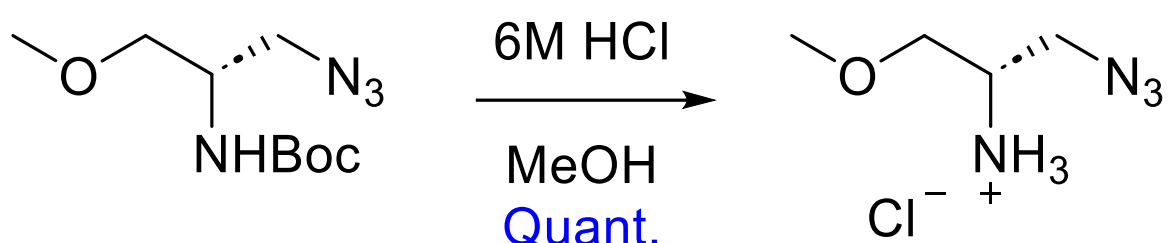

2.5

Me-ETA-S-N$_3$

(S)-1-Azido-3-methoxypropan-2-amine hydrochloride (Me-ETA-S-N$_3$)

Compound **2.5** (0.3575 g, 1.54 mmol) was added to a 100 mL round bottom flask under argon protection and cooled to 0℃. A pre-cooled solution of 6M HCl (8 mL) was added slowly to the flask and stirred for 30 minutes at room temperature. The mixture was diluted with MeOH (10 mL) and concentrated under vacuum to yield **Me-ETA-S-N$_3$** in quantitative yield as a colorless solid (0.255 g, 1.54 mmol).

^{1}H NMR 500 MHz (CD_3OD) δ 3.81-3.75 (dd, J = 5.6, 1H), 3.71-3.61 (m, 2H), 3.60-3.64 (q, J = 6.1, 1H), 3.51 (s, 1H), 3.41 (s, 3H). ^{13}C NMR (126 MHz, CD_3OD) δ 69.21, 58.25, 50.42, 49.50.

Supplementary Figures

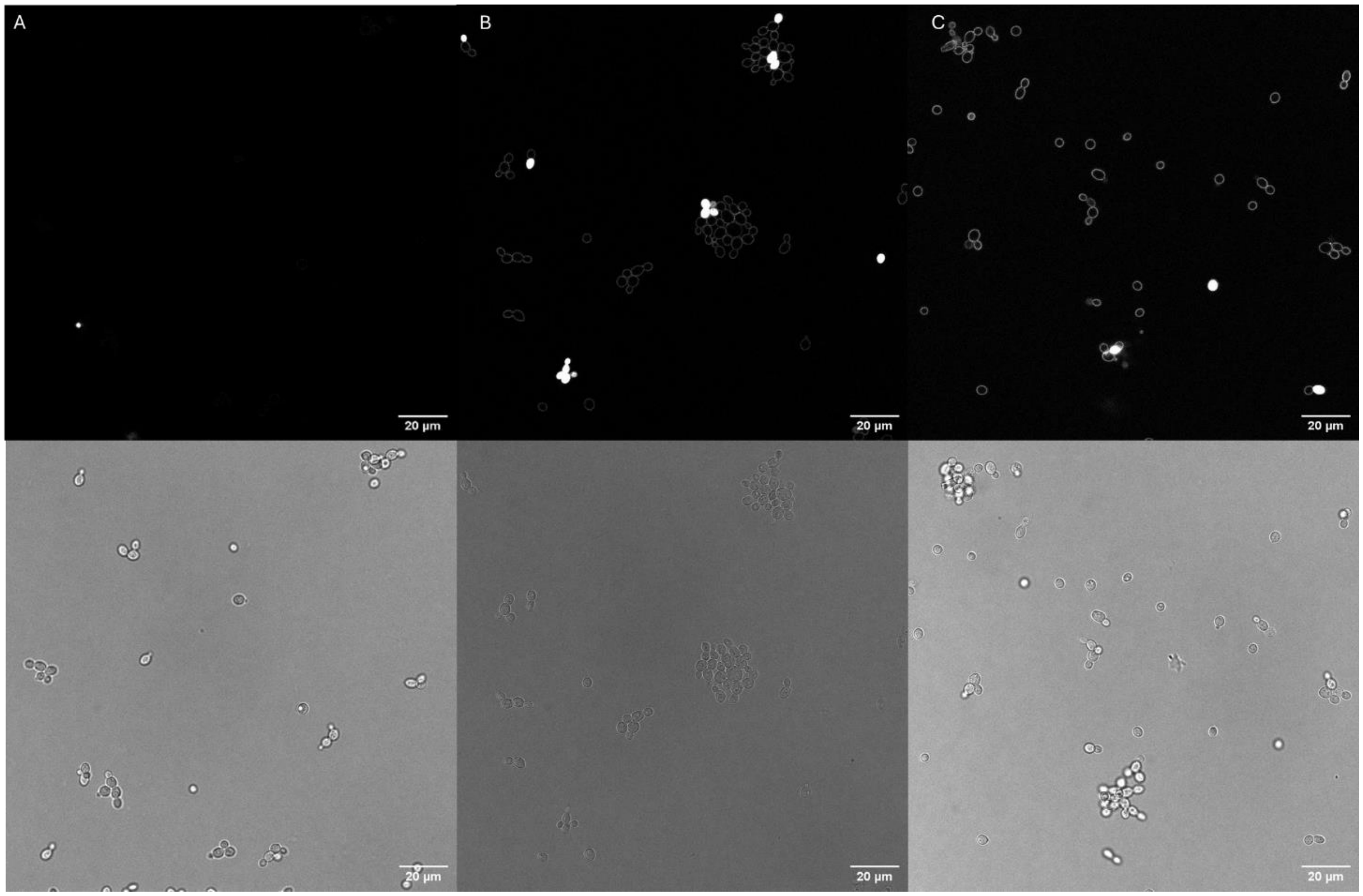

Figure S1. Bright-field and fluorescence microscopy images of *S. cerevisiae* cultured in the presence of 1 mM **ETA-R-N₃**, **ETA-R-N₃**, or water as a negative control (C, B, and A, respectively), collected at a zoom factor of 1. Cells were treated with either probe or water for 2 hours, fixed, and then exposed to 1 µM Cy3-DBCO for SPAAC labeling. Fluorescent signals were observed in cells incubated with the probe, whereas cells treated only with water displayed little to no fluorescence. Dead cells exhibited diffuse cytosolic labeling and were excluded from quantification. Bright-field images show cell positioning, and the scale bar represents 20 µm.

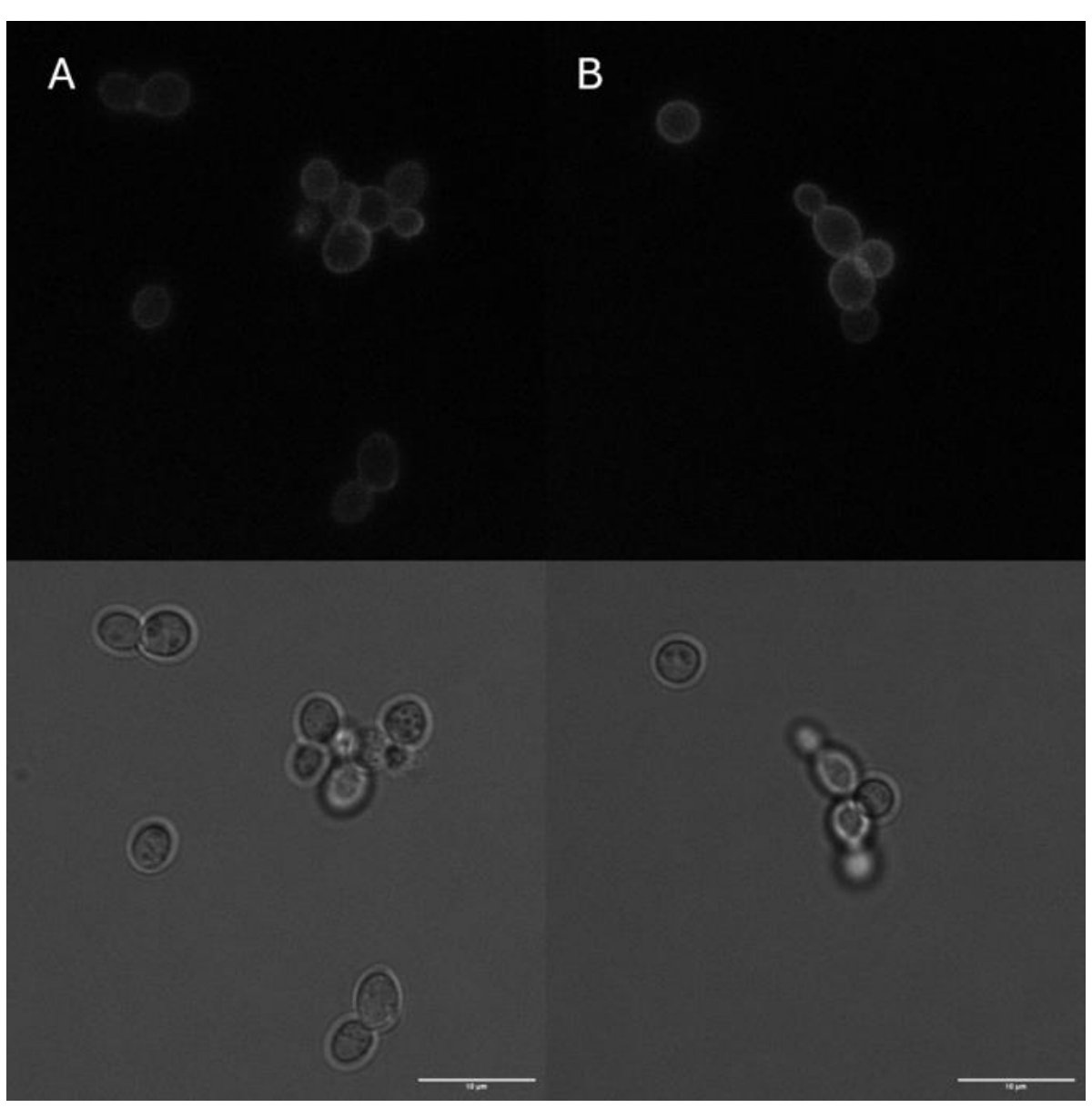

Figure S2. Bright-field and fluorescence microscopy images of *S. cerevisiae* cultured in the presence of 1 mM **Me-ETA-S-N$_3$** or **Me-ETA-R-N$_3$** collected at a zoom factor of 4 (A and B) as a z-stack of 12 images with their fluorescence maximized. Cells were treated in the same manner as in image S1, but the scale bar represents 10 µm.

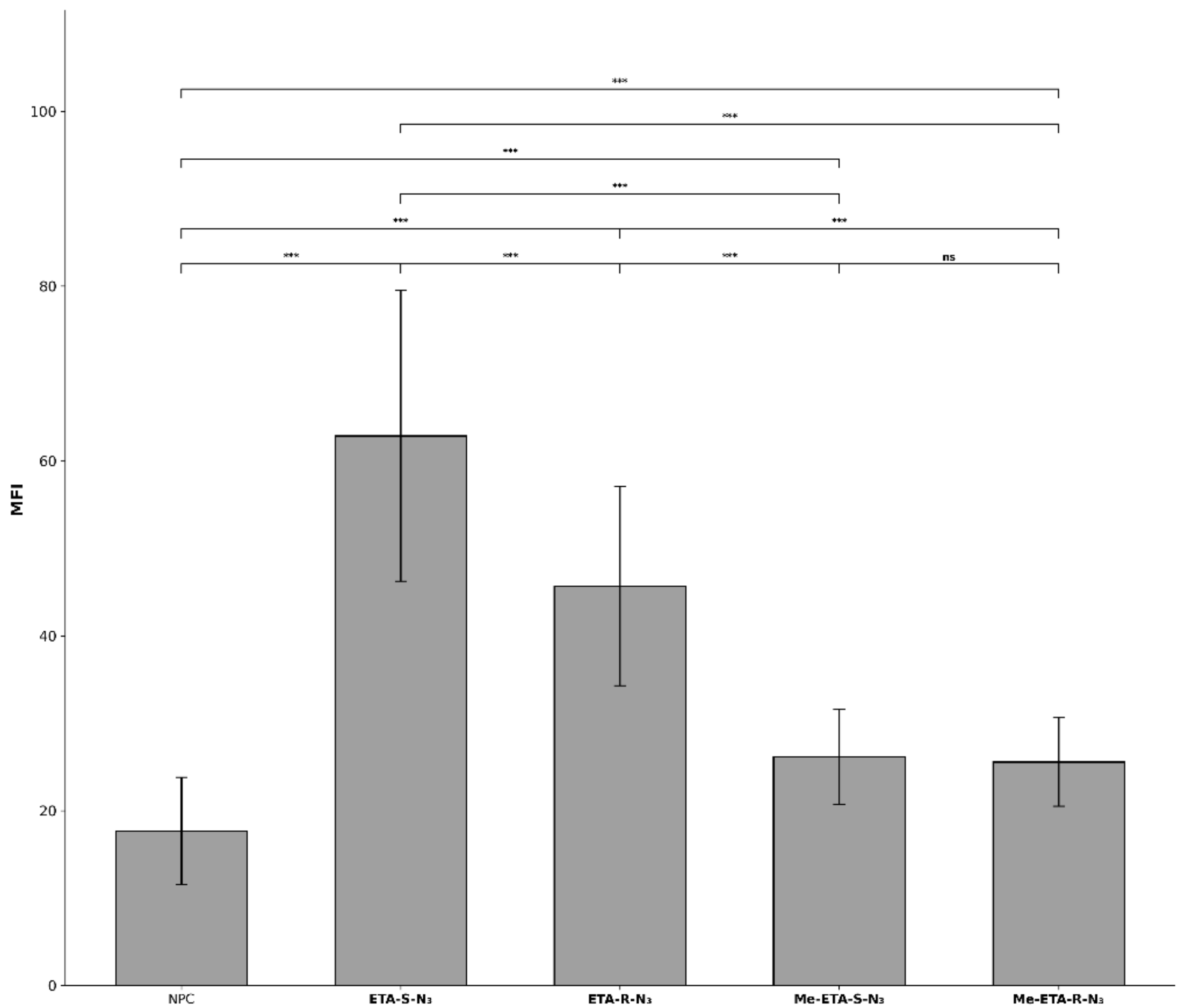

Figure S3: Mean fluorescence intensity (MFI) of no probe control (NPC), **ETA-S-N$_3$**, **ETA-R-N$_3$**, **Me-ETA-S-N$_3$**, and **Me-ETA-R-N$_3$**. Data are presented as mean ± standard deviation (SD) from triplicate measurements. Statistical significance was determined by post hoc Welch's t-test with Bonferroni correction for multiple comparisons. All pairwise comparisons were significant ($p < 0.001$, ***) except **Me-ETA-S-N$_3$** vs **Me-ETA-R-N$_3$** (ns, $p = 0.590$).

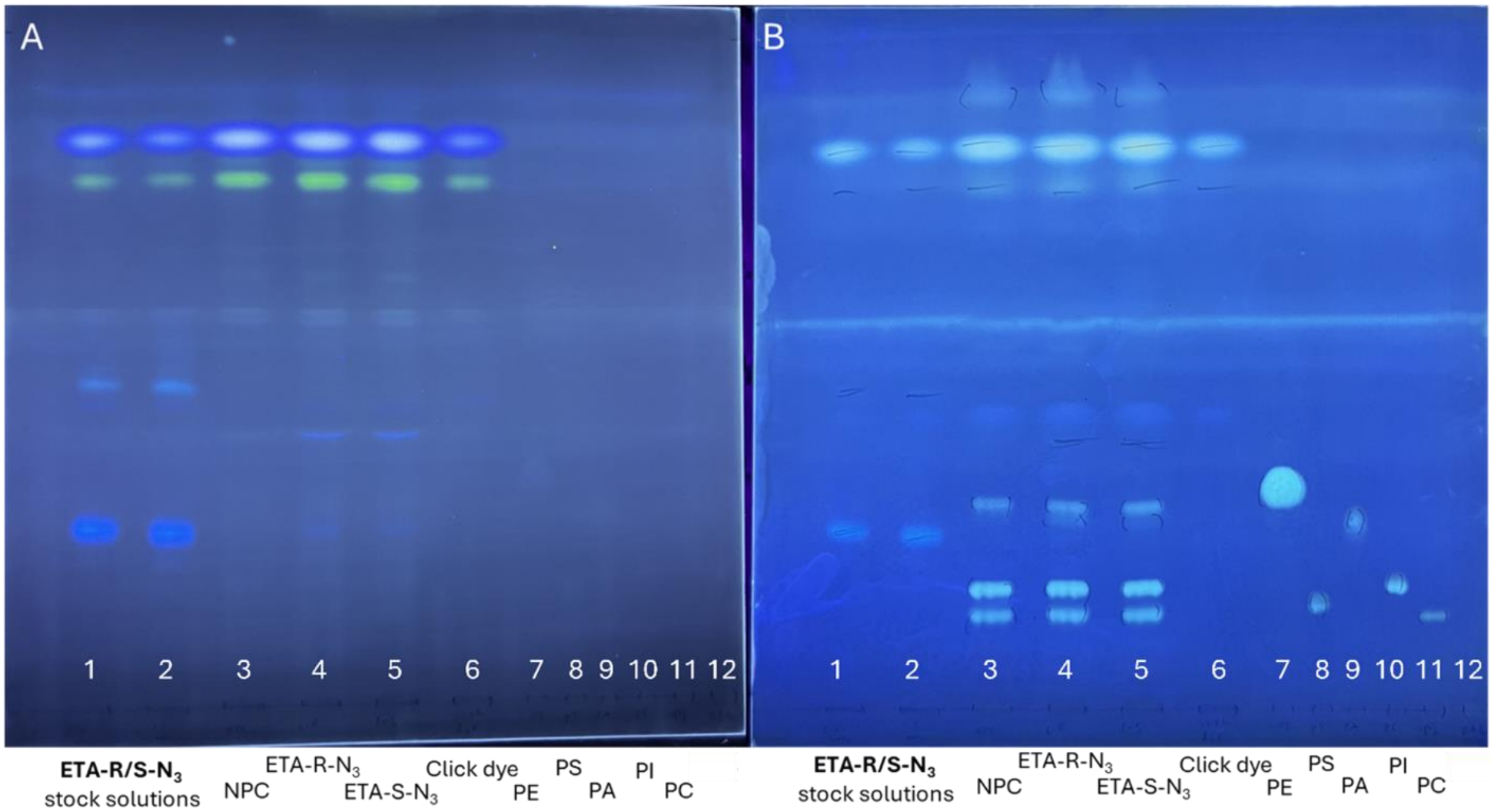

Figure S4. Unedited images of the full TLC plates imaged pertaining to Figure 5. Images were taken before (A) and after (B) primulin staining. The first 6 lanes in order from left to right are: 1mM **ETA-R-N₃** stock solution, 1mM **ETA-S-N₃**, extracted lipids from cells treated with no probe control, extracted lipids from cells treated with **ETA-R-N₃**, extracted lipids from cells treated with **ETA-S-N₃**, and the clickable dye **Naphth**. The final 6 lanes were spotted with commercially available lipid standards and are in order: PE, PS, PA, PI, PC.

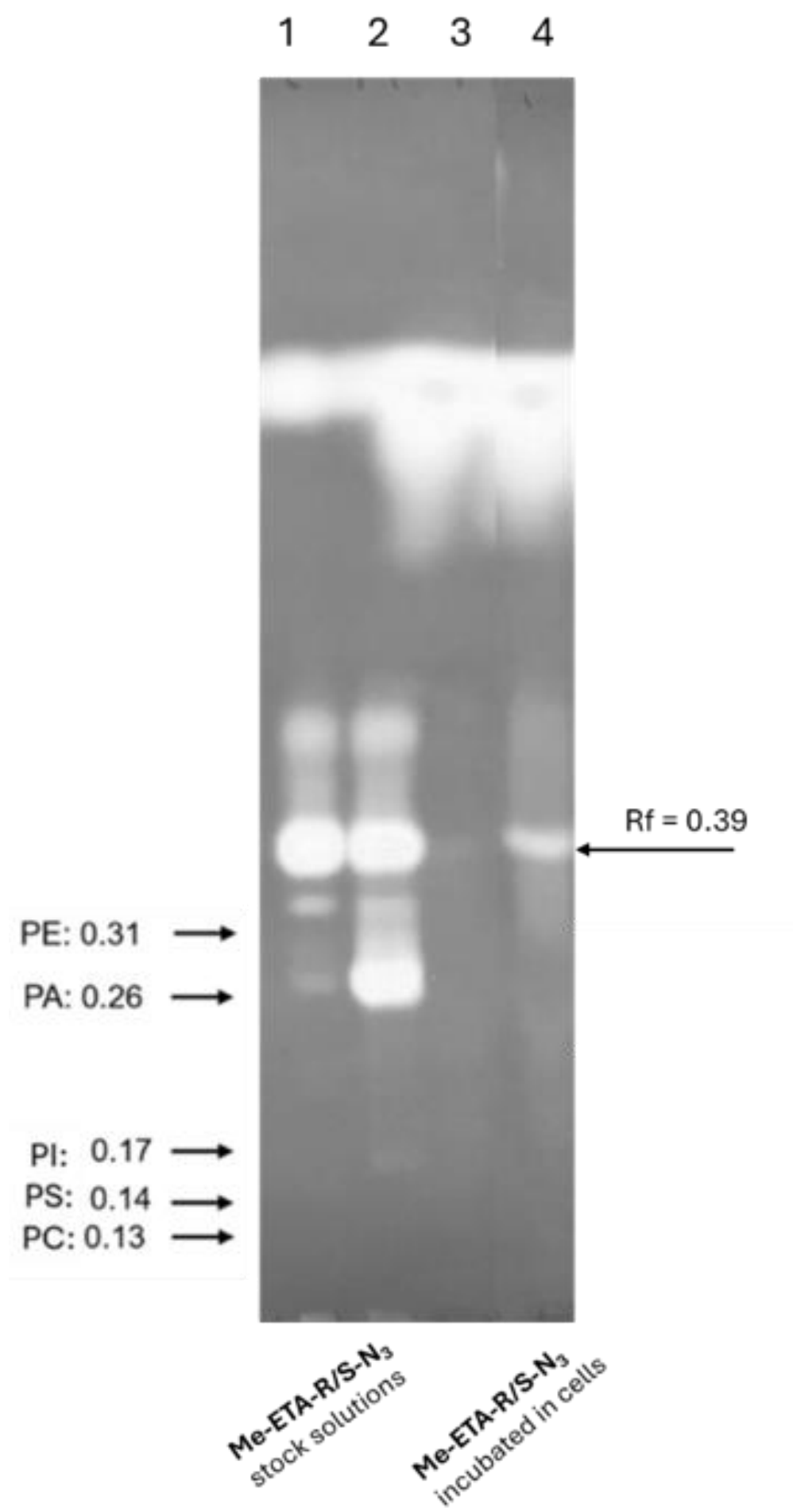

Figure S5: TLC image resulting from treatment with methylated negative control probes **Me-ETA-S-N$_3$** and **Me-ETA-R-N$_3$.** Lanes 1 and 2 are stock solutions of **Me-ETA-R-N$_3$** and **Me-ETA-S-N$_3$**, and lanes 3 and 4 are cell extracts from cells treated with the aforementioned methylated probes. Lipid standards PE, PA, PI, PS, and PC were also on the plate with their respective R_f values shown on the left side of the plate. There were no new bands observed that were comparable to those of the lipid standards. The disappearance of the new fluorescent band that was observed upon **ETA-R-N$_3$** or **ETA-S-N$_3$** treatment when using methylated negative controls further supports labeling by the reported probes.

Lipid Species	Adduct	Observed mass	Expected mass	Error (ppm)	RT	Native RT	RT difference	Native lipid	Adduct	Isobar info
(28:1)PEmN3	H-	687.446	687.4467	-1.018260761	12.72	14.01	-1.29	(28:1)PE	H	
(30:1)PDMEmN3	H-	743.5091	743.5093	-0.268994618	15.46	13.85	1.61	(30:1)PE	H	
(30:2)PEmN3	H-	713.4617	713.4624	-0.981130891	12.92	12.7	0.22	(30:2)PE	H	
(30:2)PDMEmN3	H-	741.4929	741.4937	-1.0789033	14.25	12.7	1.55	(30:2)PE	H	
(32:1)PDMEmN3	H-	771.54	771.5406	-0.777664844	17.12	15.4	1.72	(32:1)PE	H	
(32:2)PDMEmN3	H-	769.5252	769.525	0.259900588	15.73	13.98	1.75	(32:2)PE	H	
(34:2)PMMEmN3	H-	783.5391	783.5406	-1.914387078	15.61	15.48	0.13	(34:2)PE	H	
(34:2)PDMEmN3	H-	797.556	797.5563	-0.376148994	17.19	15.48	1.71	(34:2)PE	H	
(28:1)PDMEmN3	H+	717.4944	717.4926	2.508736675	14.1	12.43	1.67	(28:1)PE	H	
(30:1)PDMEmN3	H+	745.5227	745.5239	-1.609606345	15.63	13.42	2.21	(30:1)PE	H	
(30:2)PDMEmN3	H+	743.5083	743.5082	0.134497508	14.29	12.71	1.58	(30:2)PE	H	
(32:1)PDMEmN3	H+	773.5552	773.5552	0	17.01	12.71	4.3	(32:1)PE	H	
(32:2)PDMEmN3	H+	771.5391	771.5395	-0.518443968	15.73	15.39	0.34	(32:2)PE	H	
(34:1)PDMEmN3	H+	801.5872	801.5865	0.8732682	18.63	13.99	4.64	(34:1)PE	H	Isobar with (36:1)PEmN3
(34:2)PMMEmN3	H+	785.5561	785.5552	1.145686516	15.65	15.49	0.16	(34:2)PE	H	
(36:1)PEmN3	H+	801.5872	801.5865	0.8732682	18.63	15.44	3.19	(36:1)PE	H	Isobar with (34:1)PDMEmN3
(36:2)PEmN3	H+	799.5699	799.5708	-1.125603886	17.18	14.06	3.12	(36:2)PE	H	

Table S1. Detected mass peaks during LCMS experiments corresponding to labeled PE products from cells incubated with **ETA-R-N$_3$** and **ETA-R-N$_3$**. Azide-labeled phospholipids were detected as either $[M+H]^+$ or $[M-H]^-$ adducts. Labeled PE species of various tail lengths were observed in addition to PMME and PDME, demonstrating the ability to detect lipids during the different headgroup modifications to form PC.

A. Negative Ion Detection

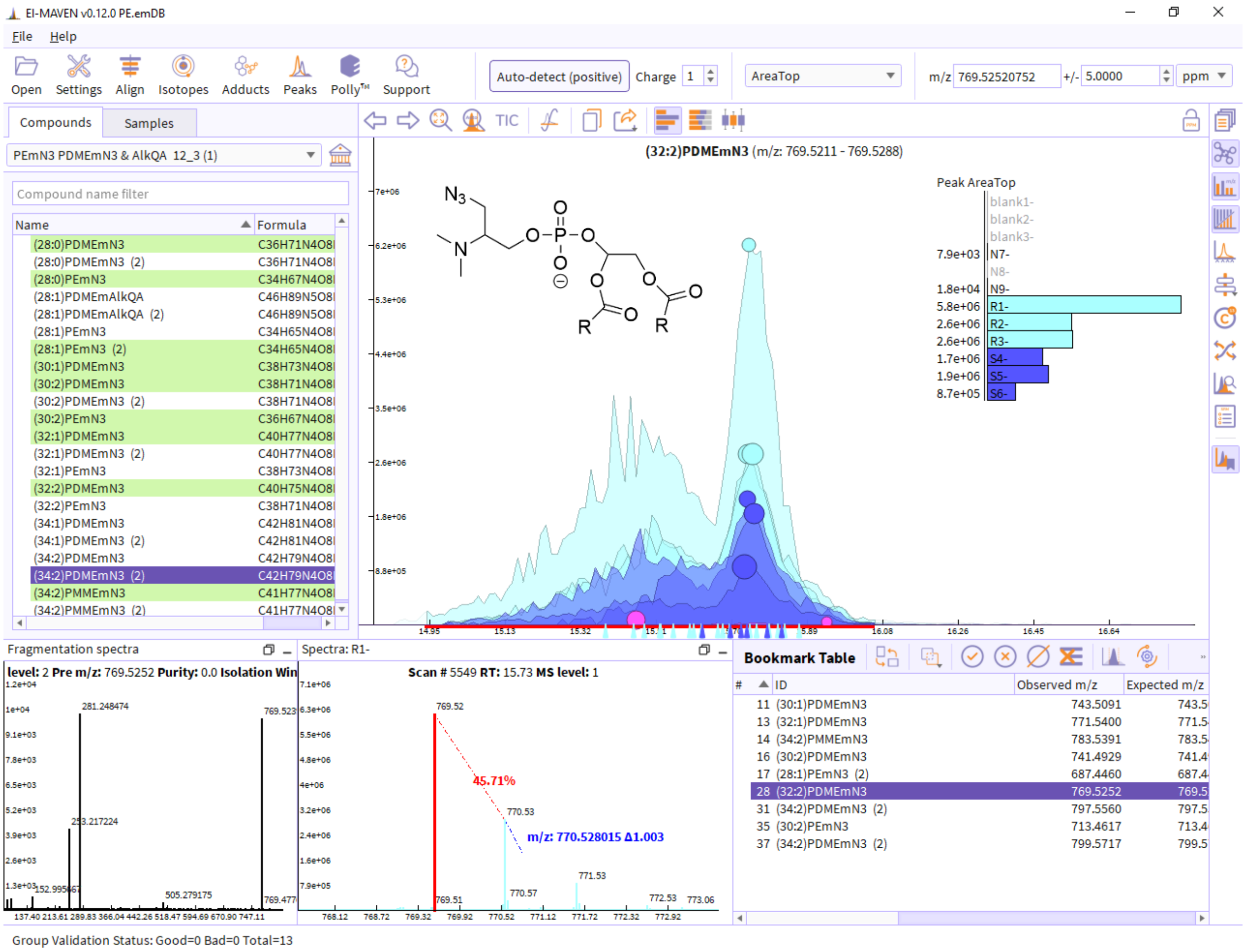

B. Positive Ion Detection

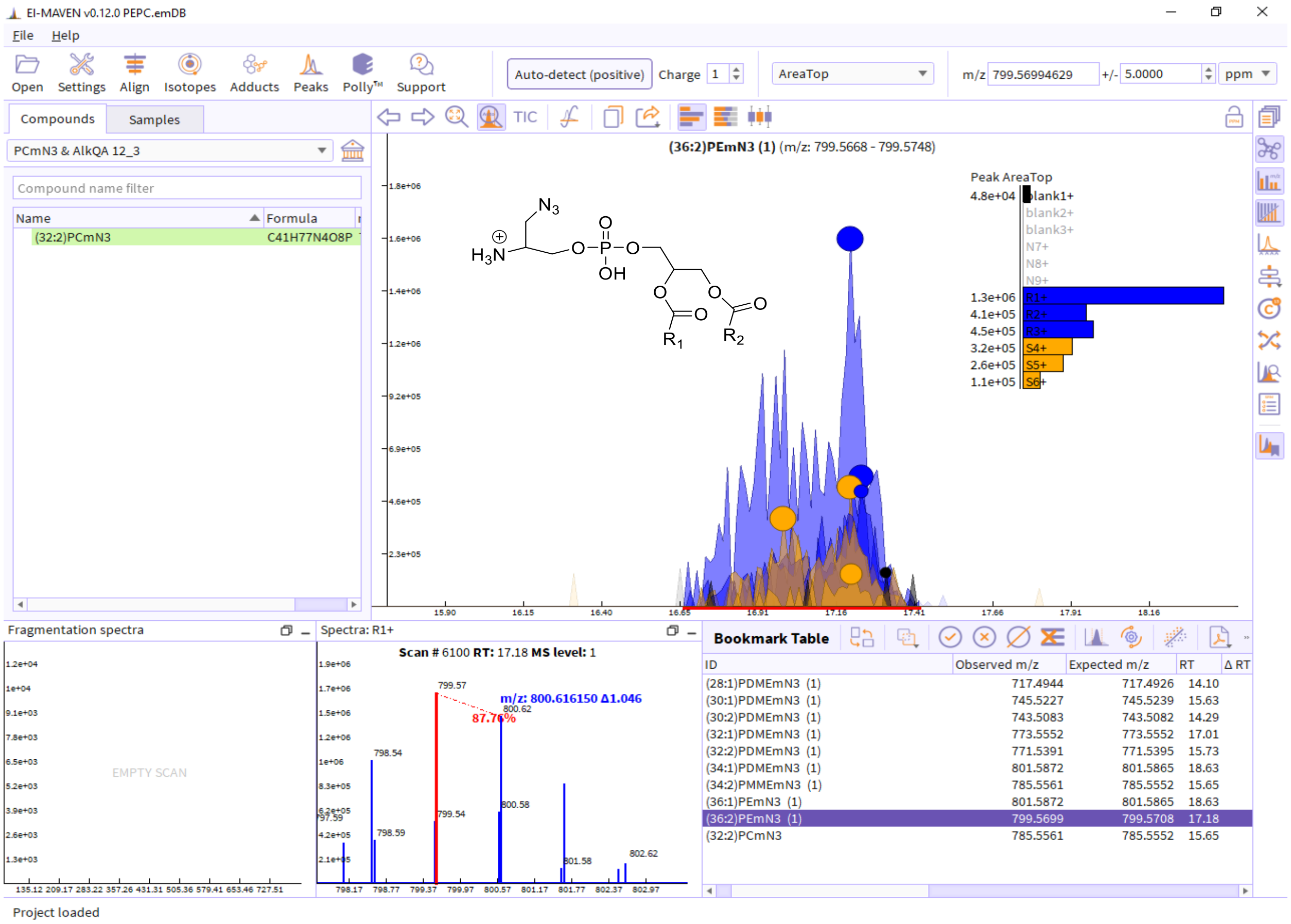

Figure S6. Representative mass spectra of the lipid species detected by liquid chromatography mass spectrometry (LCMS). Image A showing the detection of (32:2) PDMEmN$_3$ in negative mode while image B shows the detection of the lipid species (36:2) PEmN$_3$ in positive mode. For both images, black peaks indicate blank samples and pink samples indicated no probe control samples. For Image A, light blue peaks were cell extracts taken from cells incubated with probe **ETA-R-N$_3$**, and dark blue peaks were cell extracts taken from cells incubated with probe **ETA-S-N$_3$**. For Image B, blue peaks were cell extracts taken from cells incubated with probe **ETA-R-N$_3$**, and orange peaks were cell extracts taken from cells incubated with probe **ETA-S-N$_3$**. The detections were done in triplicate, which is shown by the 3 different bars of each color.

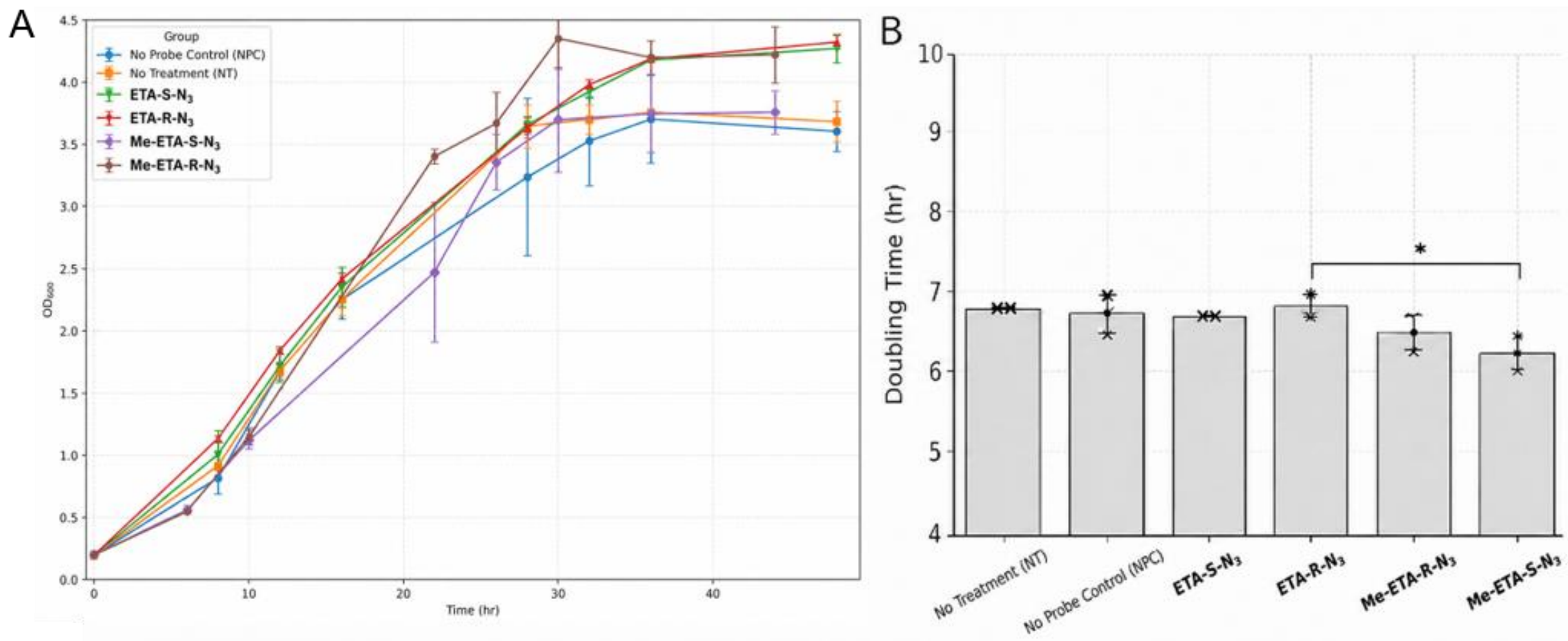

Figure S7. Panel A shows a growth curve of *S. cerevisiae* cells incubated with water (NPC), no treatment, **ETA-R-N$_3$**, **ETA-S-N$_3$**, **Me-ETA-R-N$_3$**, or **Me-ETA-S-N$_3$**. Pointwise one-way ANOVA with Tukey's HSD indicated that Me-ETA-R-N$_3$ was higher than Me-ETA-S-N$_3$ at 22 h ($p = 0.0459$) and 44 h ($p = 0.0483$); however, these differences were not significant after Holm correction across the tested timepoints. Panel B shows doubling times calculated from the exponential growth phase and shown as mean ± SD. Statistical analysis using Welch's ANOVA with Games-Howell post-hoc analysis identified a significant difference between **ETA-R-N$_3$** and **Me-ETA-S-N$_3$** only, $p = 0.0495$. *$p < 0.05$. All other comparisons were not significant.

Supplementary References

[1] Hanson, B. A.; Lester, R. L., The extraction of inositol-containing phospholipids and phosphatidylcholine from Saccharomyces cerevisiae and Neurospora crassa. *Journal Of Lipid Research* **1980**, 21 (3), 309-315.

[2] Bumpus, T. W.; Baskin, J. M., Clickable substrate mimics enable imaging of phospholipase D activity. *ACS Central Science* **2017**, 3 (10), 1070-1077.

[3] Woodall, B. M.; Harp, J. R.; Brewer, W. T.; Tague, E. D.; Campagna, S. R.; Fozo, E. M., Enterococcus faecalis readily adapts membrane phospholipid composition to environmental and genetic perturbation. *Frontiers in Microbiology* **2021**, 12, 616045.

Spectra of Synthetic Compounds

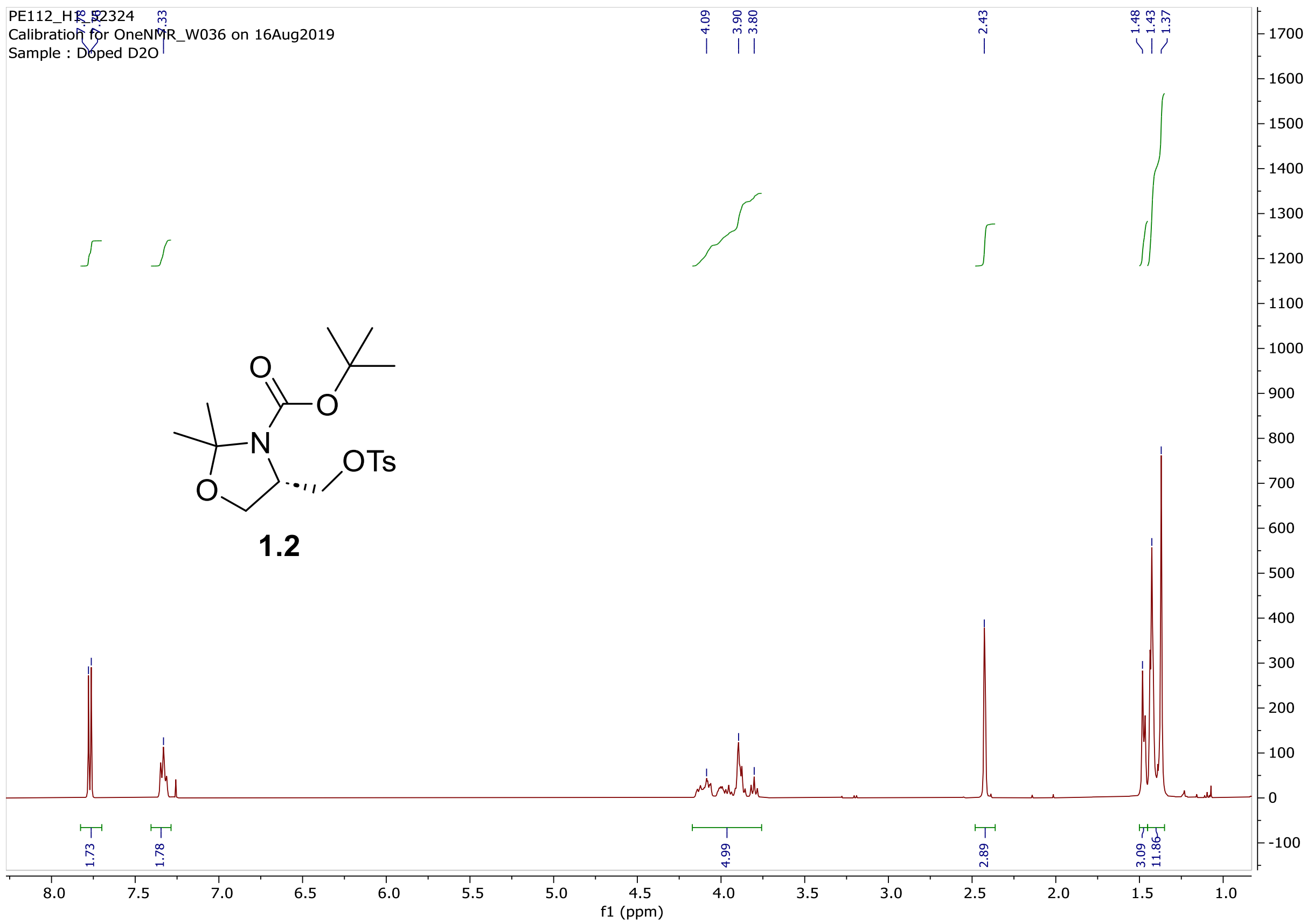

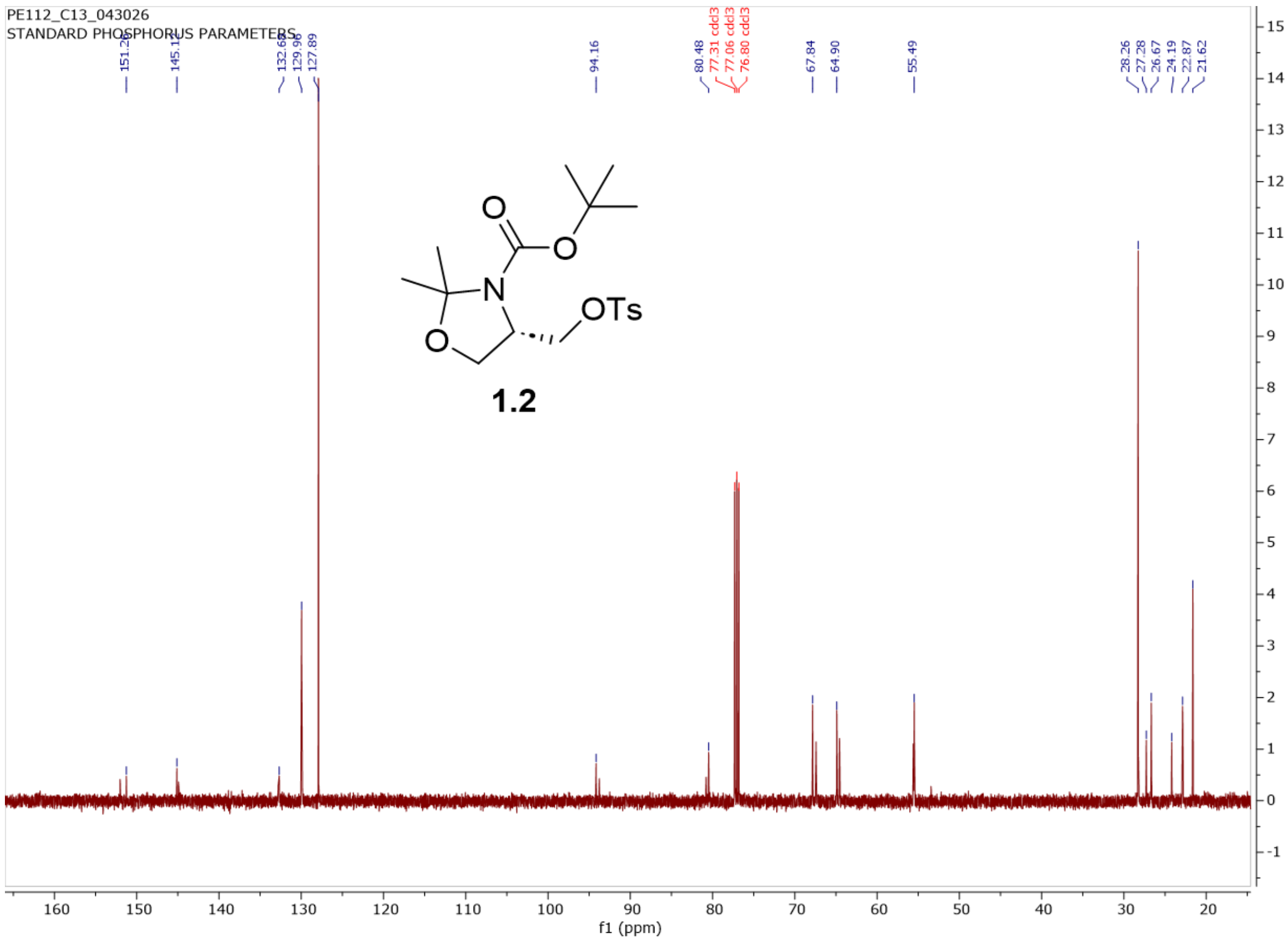
PE112_C13_043026
STANDARD PHOSPHORUS PARAMETERS
151.2
145.12
132.68
129.96
127.89
94.16
80.48
77.31 cdcl3
77.06 cdcl3
76.80 cdcl3
67.84
64.90
55.49
28.26
27.28
26.67
24.19
22.87
21.62
OTs
1.2
f1 (ppm)

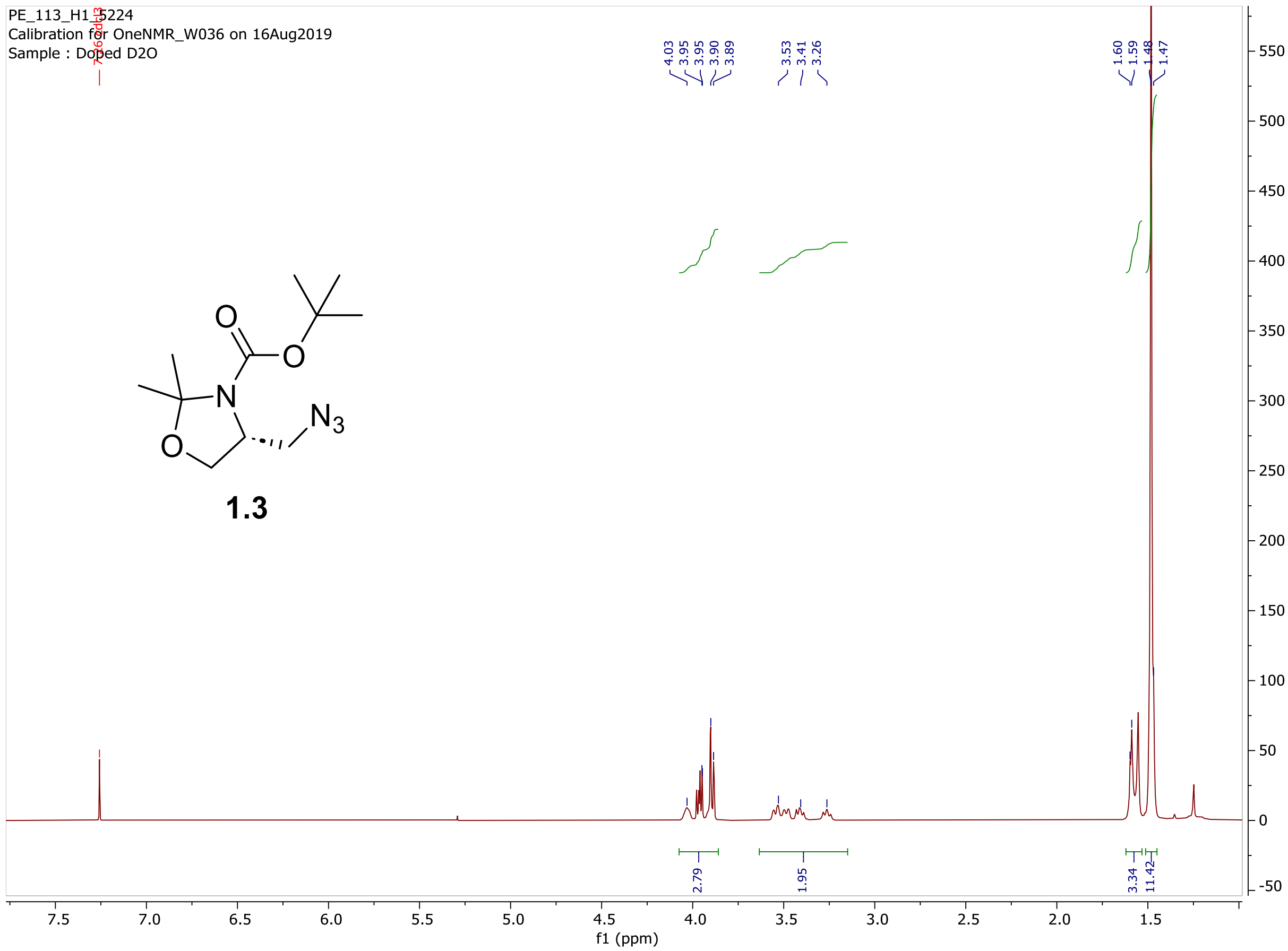
PE_113_H1_5224
Calibration for OneNMR_W036 on 16Aug2019
Sample : Doped D2O
4.03
3.95
3.95
3.90
3.89
3.53
3.41
3.26
1.60
1.59
1.48
1.47
1.3
2.79
1.95
3.34
11.42
f1 (ppm)

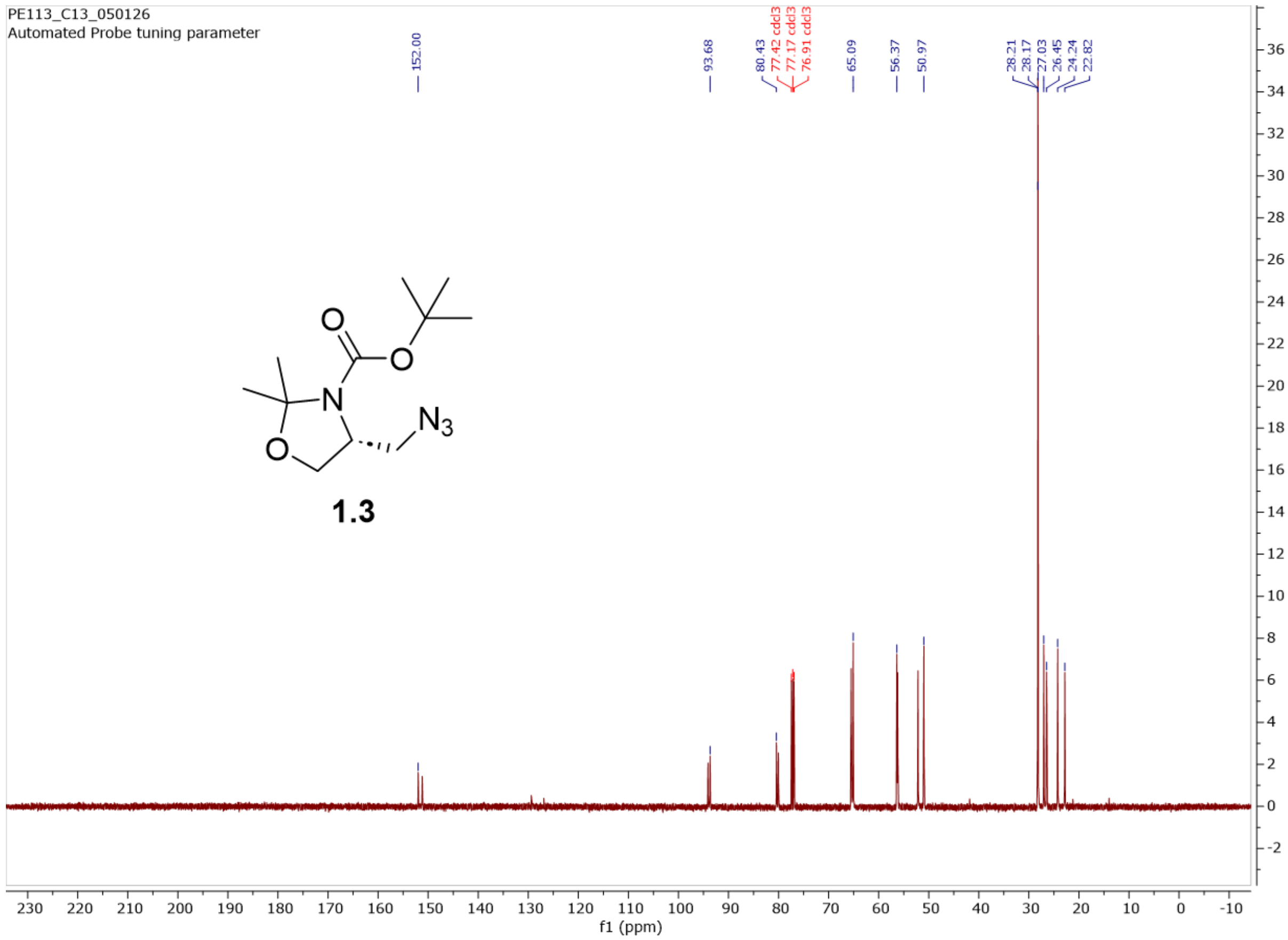
PE113_C13_050126
Automated Probe tuning parameter
152.00
93.68
80.43
77.42 cdd3
77.17 cdd3
76.91 cdd3
65.09
56.37
50.97
28.21
28.17
27.03
26.45
24.24
22.82
O
O
N
N3
O
1.3
f1 (ppm)

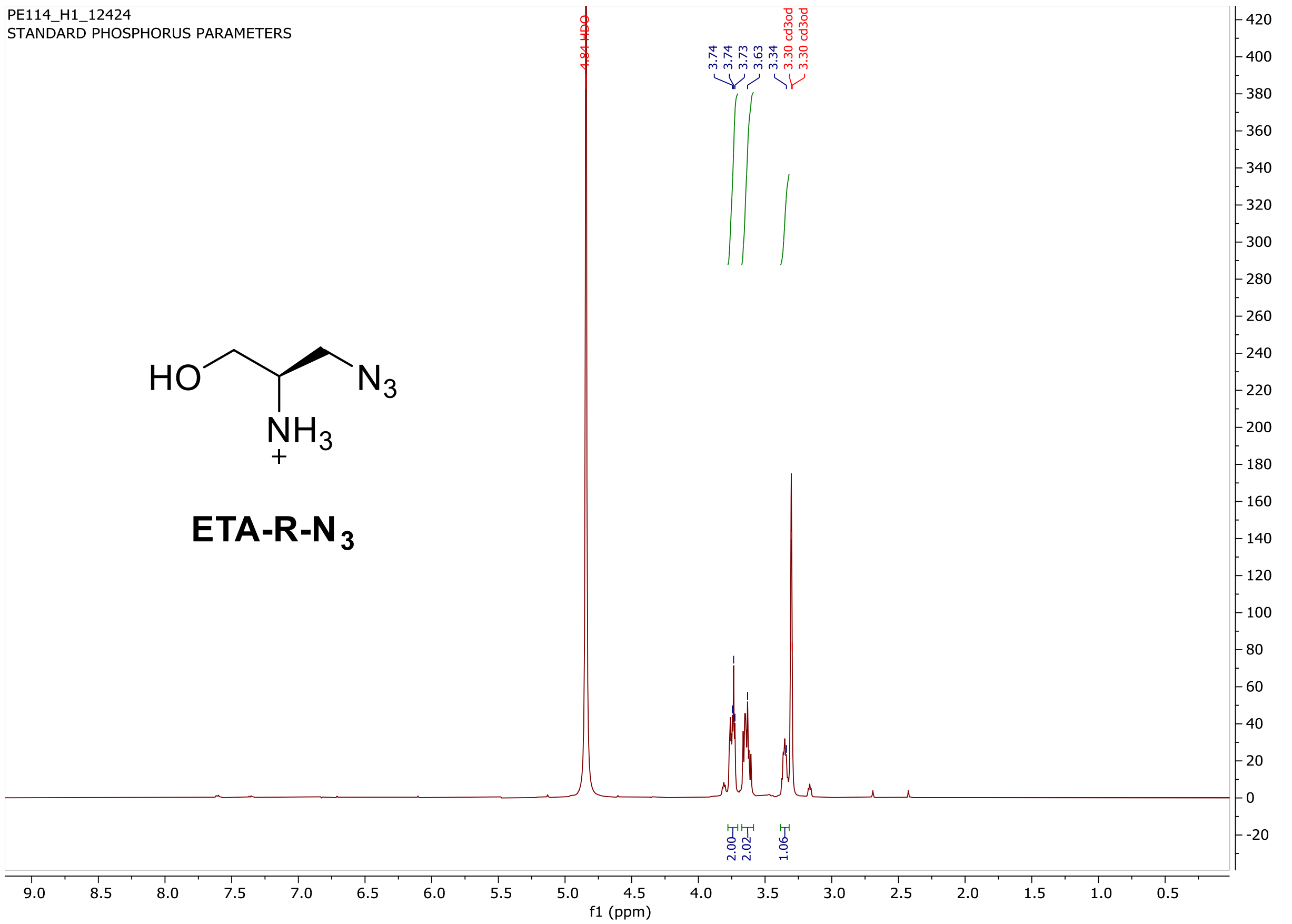
PE114_H1_12424
STANDARD PHOSPHORUS PARAMETERS
4.84 HDO
3.74
3.74
3.73
3.63
3.34
3.30 cd3od
3.30 cd3od
HO
N3
NH3
+
2.00
2.02
1.06
f1 (ppm)

ETA-R-N$_3$

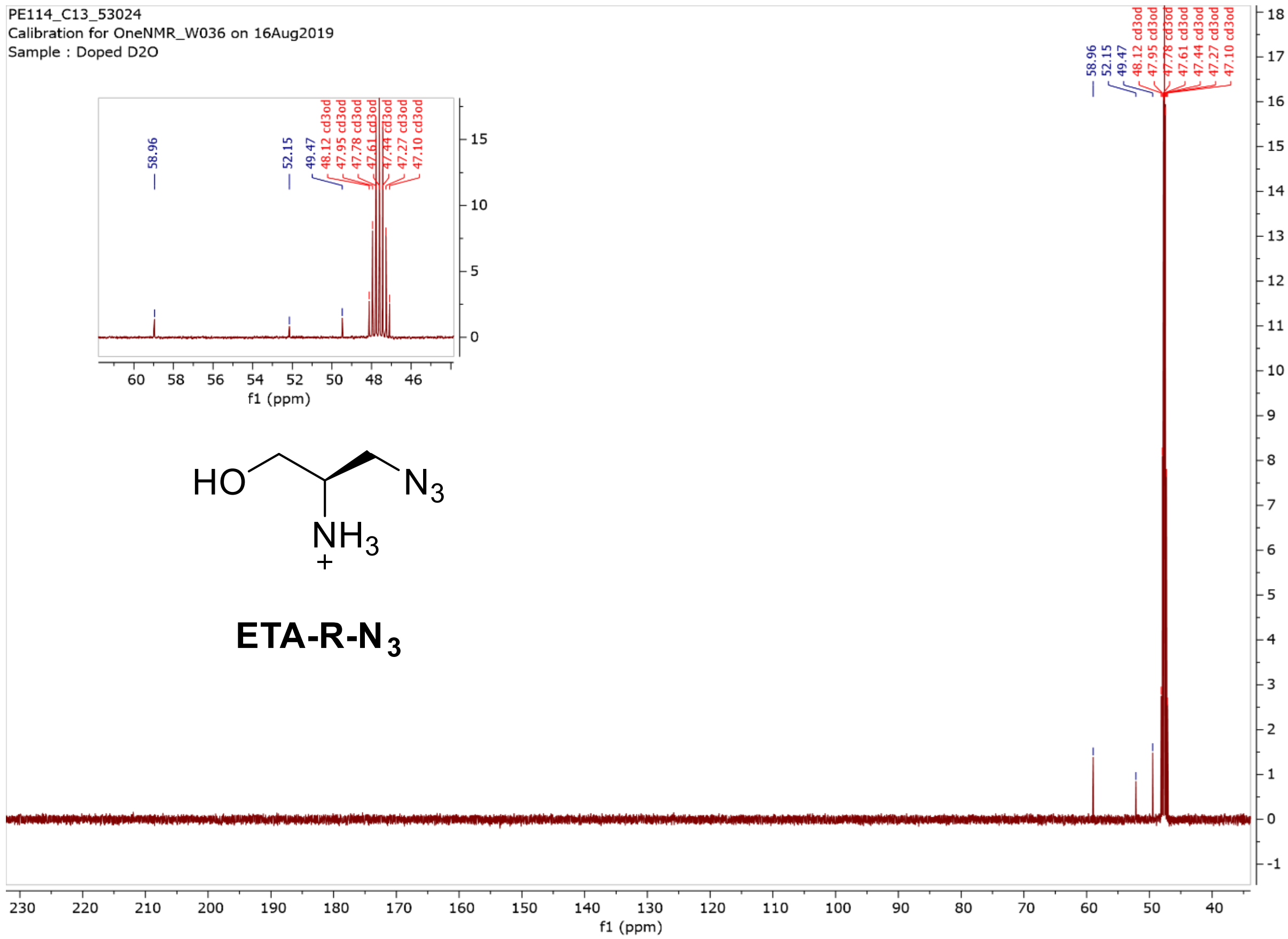
PE114_C13_53024
Calibration for OneNMR_W036 on 16Aug2019
Sample : Doped D2O
58.96
52.15
49.47
48.12 cd3od
47.95 cd3od
47.78 cd3od
47.61 cd3od
47.44 cd3od
47.27 cd3od
47.10 cd3od
f1 (ppm)
HO
N3
NH3
+
ETA-R-N3

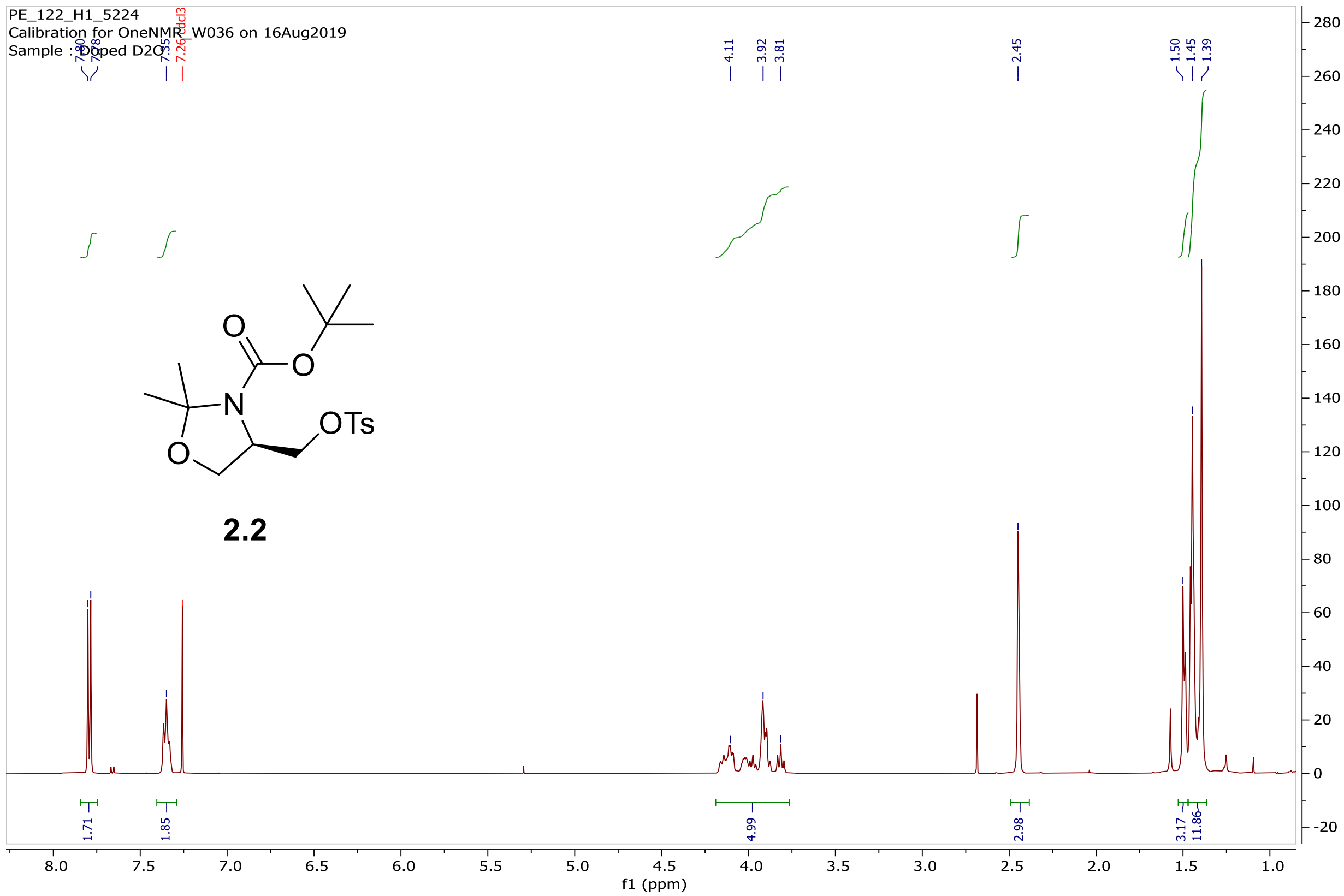

PE_122_H1_5224
Calibration for OneNMR_W036 on 16Aug2019
Sample : Doped D2O
OTs
f1 (ppm)

2.2

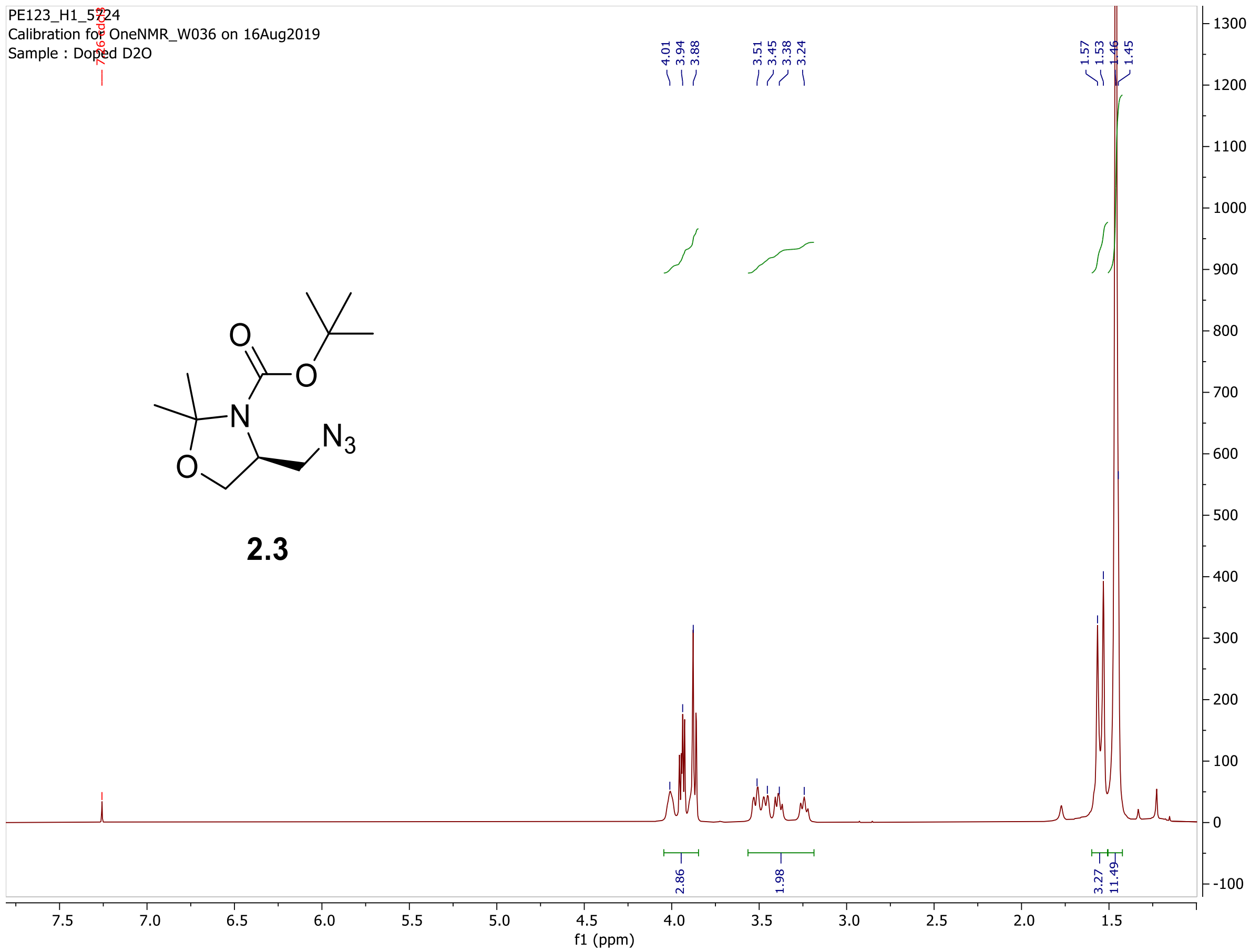
PE123_H1_5724
Calibration for OneNMR_W036 on 16Aug2019
Sample : Doped D2O
7.26
4.01
3.94
3.88
3.51
3.45
3.38
3.24
1.57
1.53
1.46
1.45
O
O
N
O
N3
2.3
2.86
1.98
3.27
11.49
f1 (ppm)

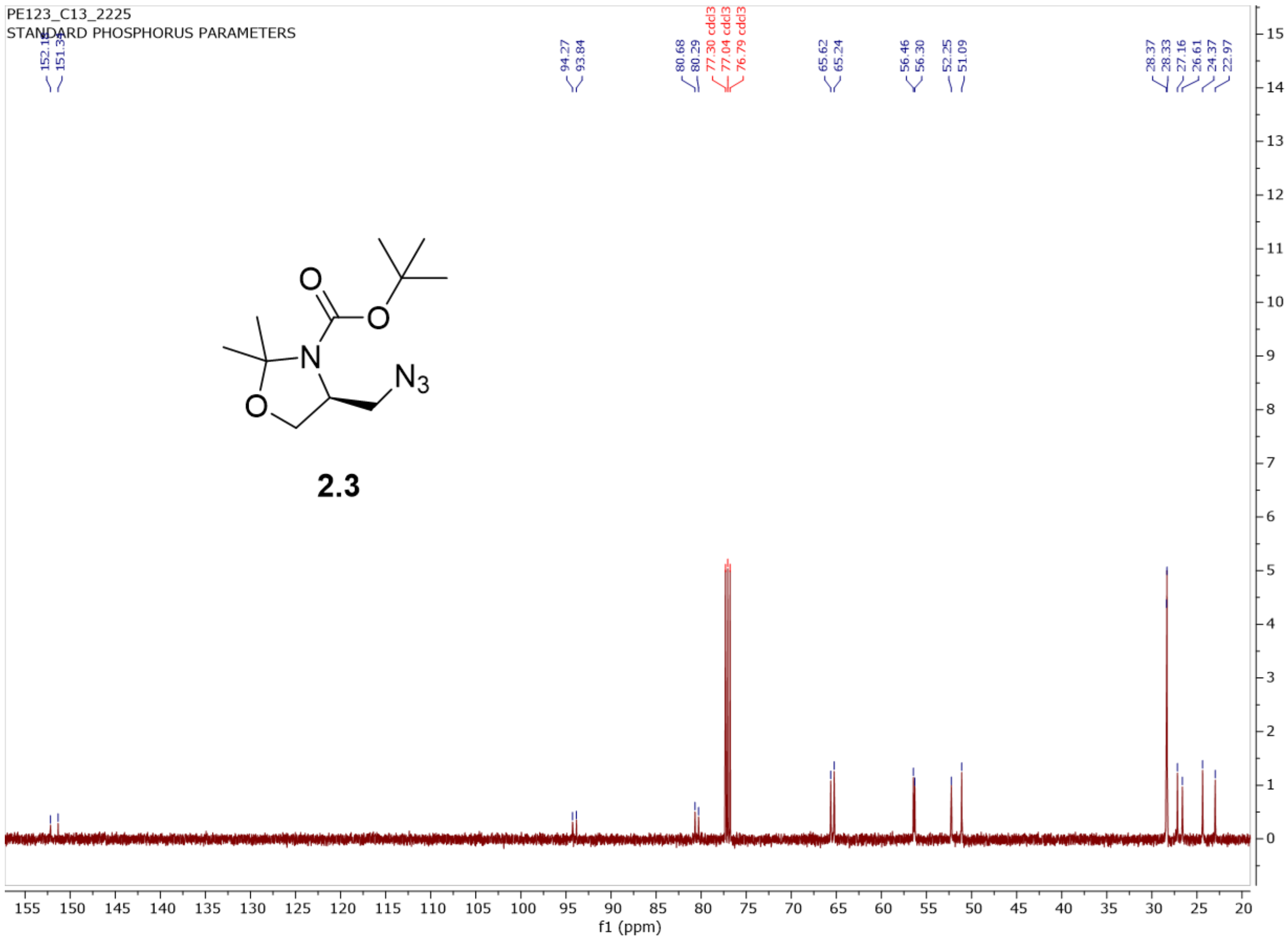
PE123_C13_2225
STANDARD PHOSPHORUS PARAMETERS
152.1
151.3
94.27
93.84
80.68
80.29
77.30 cdcl3
77.04 cdcl3
76.79 cdcl3
65.62
65.24
56.46
56.30
52.25
51.09
28.37
28.33
27.16
26.61
24.37
22.97
f1 (ppm)

2.3

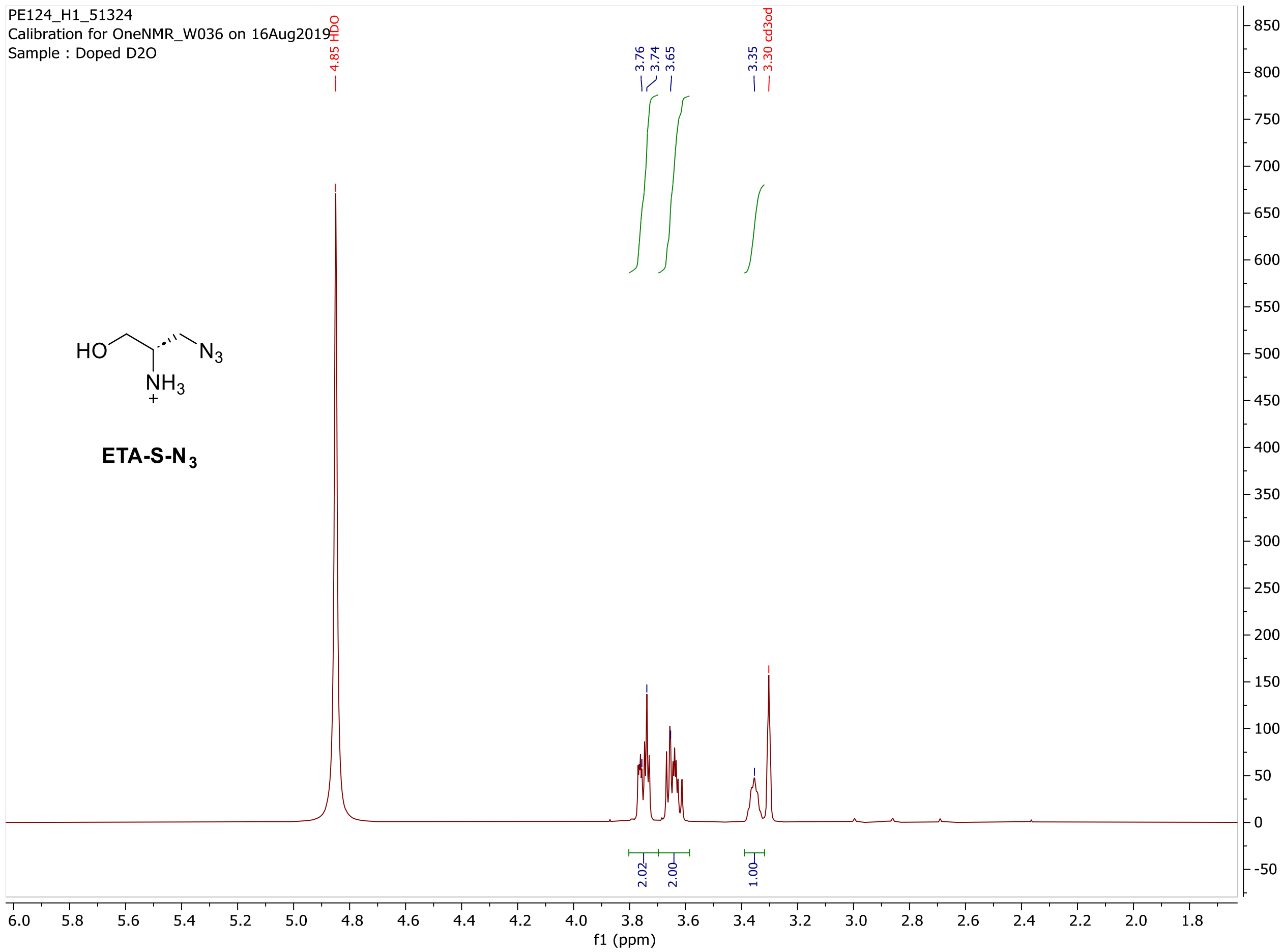

PE124_H1_51324
Calibration for OneNMR_W036 on 16Aug2019
Sample : Doped D2O
4.85 HDO
3.76
3.74
3.65
3.35
3.30 cd3od
HO
N3
NH3
+
ETA-S-N3
2.02
2.00
1.00
f1 (ppm)

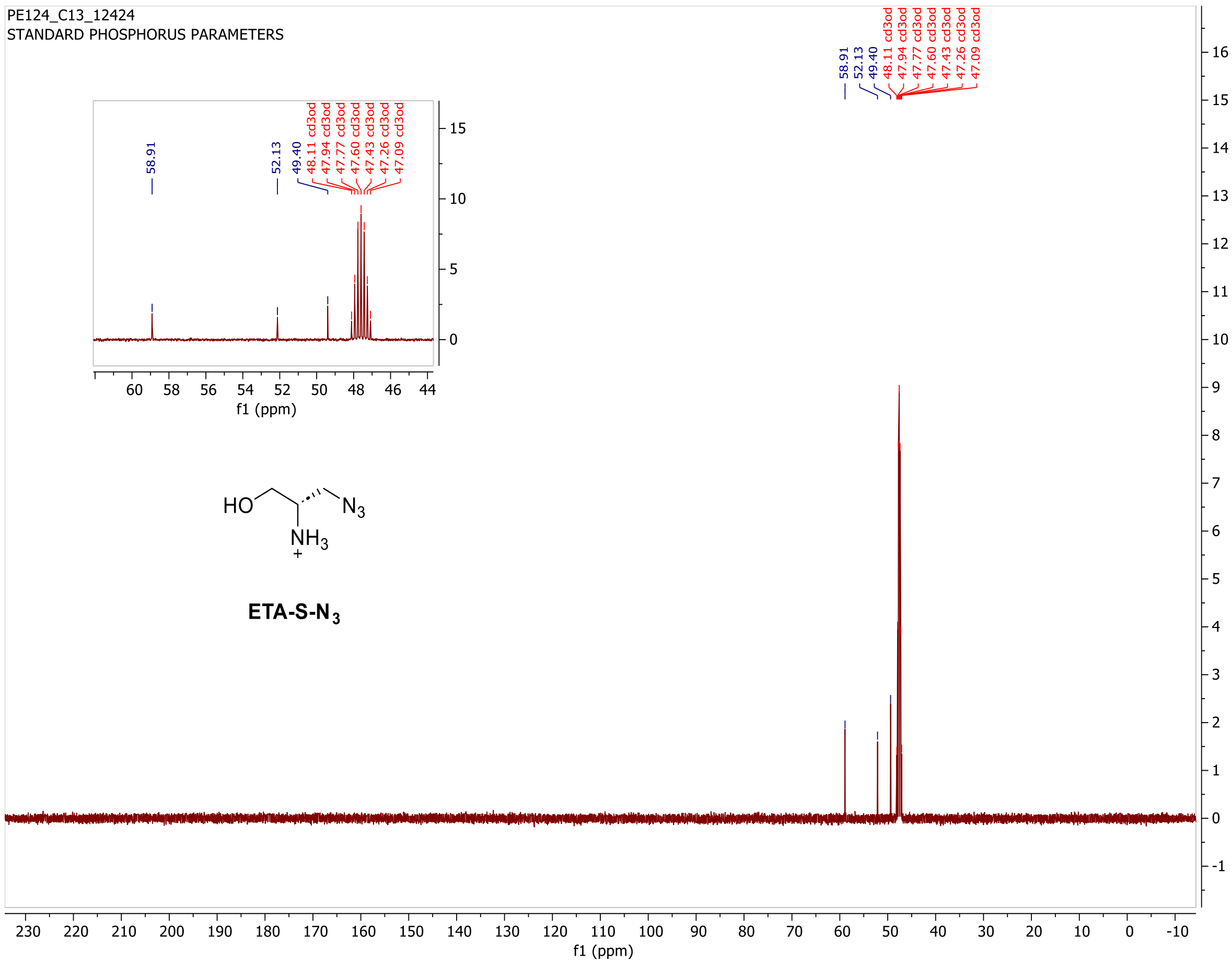
PE124_C13_12424
STANDARD PHOSPHORUS PARAMETERS
58.91
52.13
49.40
48.11 cd3od
47.94 cd3od
47.77 cd3od
47.60 cd3od
47.43 cd3od
47.26 cd3od
47.09 cd3od
f1 (ppm)
HO
N3
NH3
+

ETA-S-N$_3$

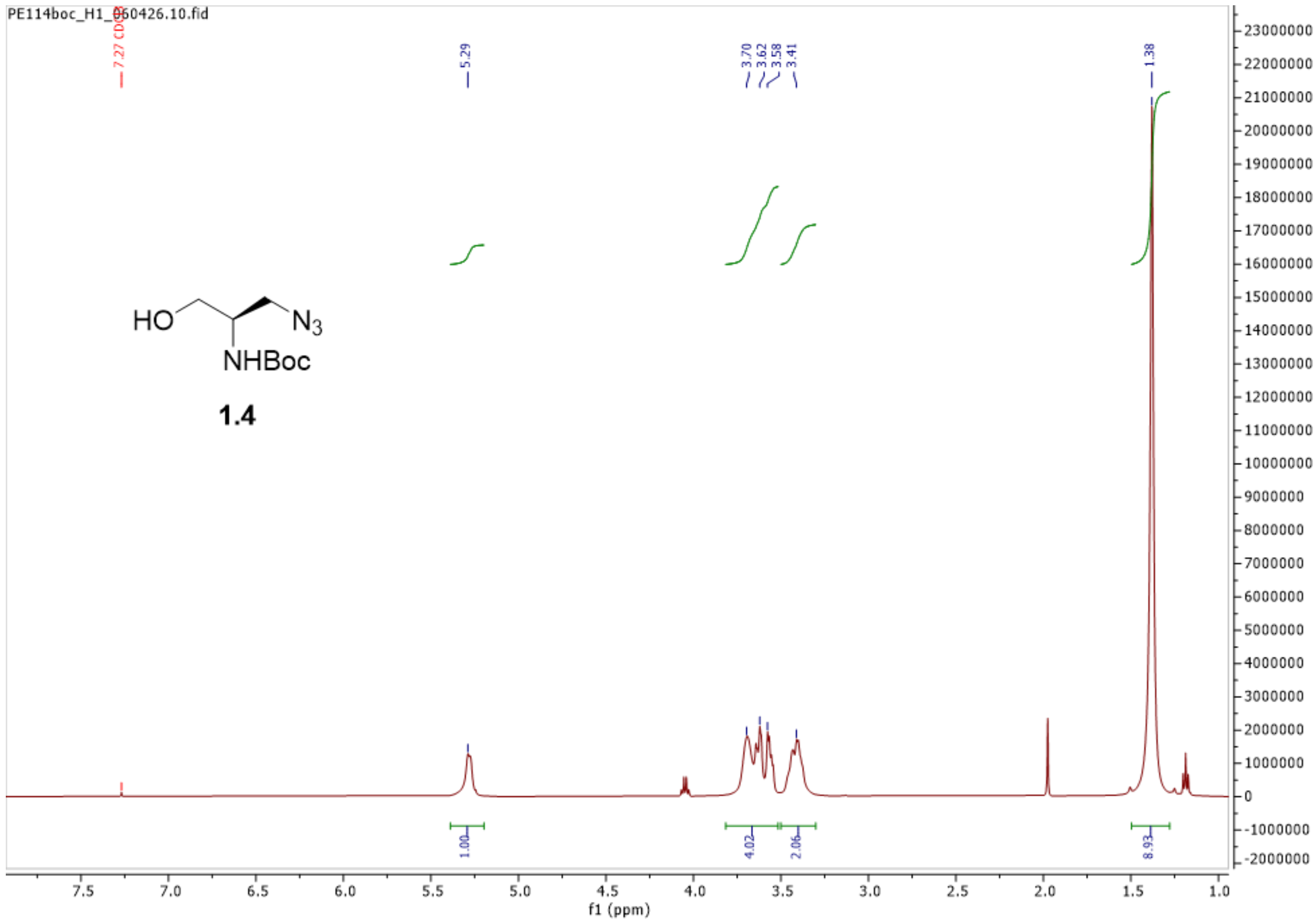
PE114boc_H1_060426.10.fid
7.27 CDCl3
5.29
3.70
3.62
3.58
3.41
1.38
HO
N3
NHBoc
1.4
1.00
4.02
2.06
8.93
f1 (ppm)

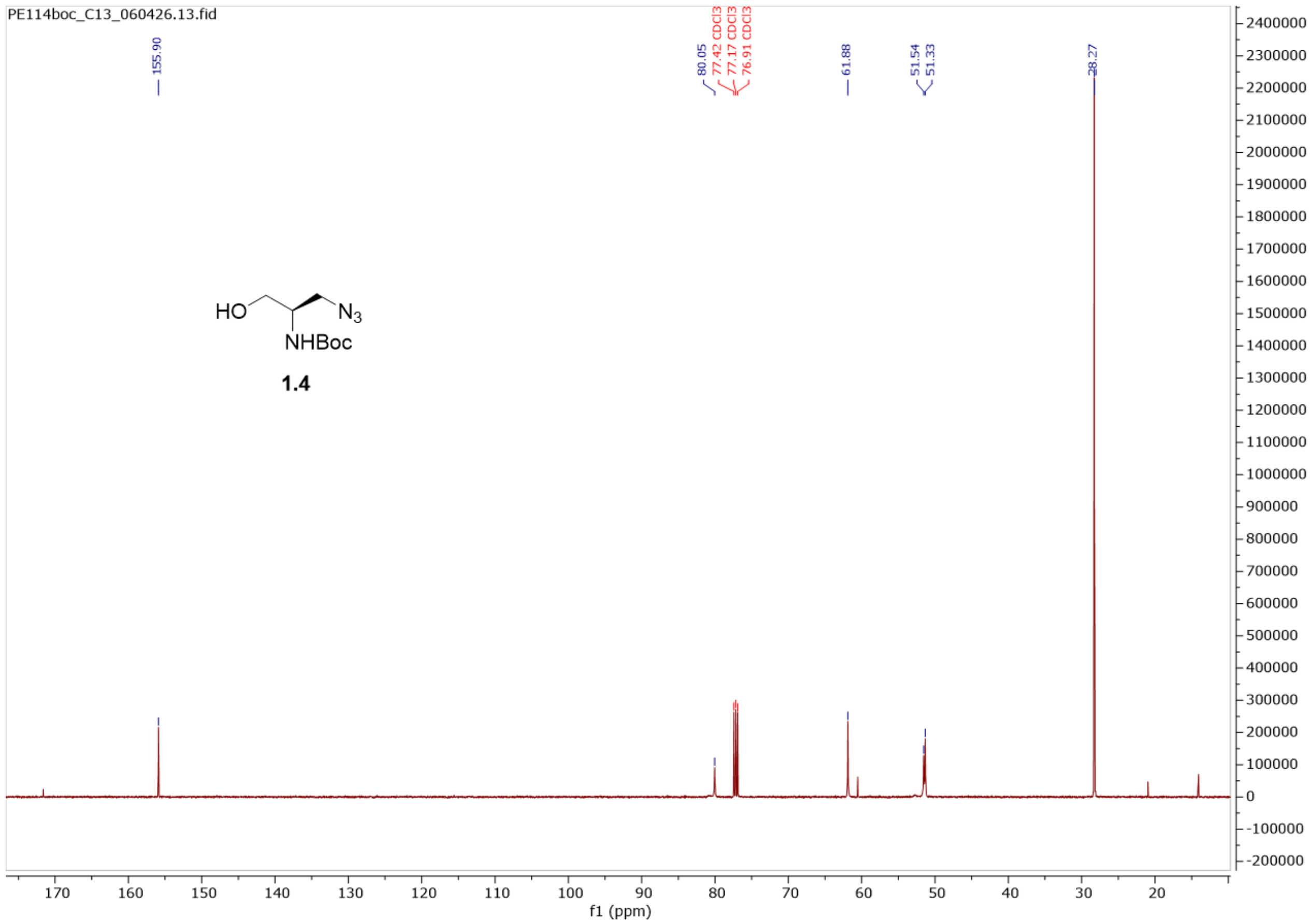
PE114boc_C13_060426.13.fid
155.90
80.05
77.42 CDCl3
77.17 CDCl3
76.91 CDCl3
61.88
51.54
51.33
28.27
HO
N3
NHBoc
f1 (ppm)

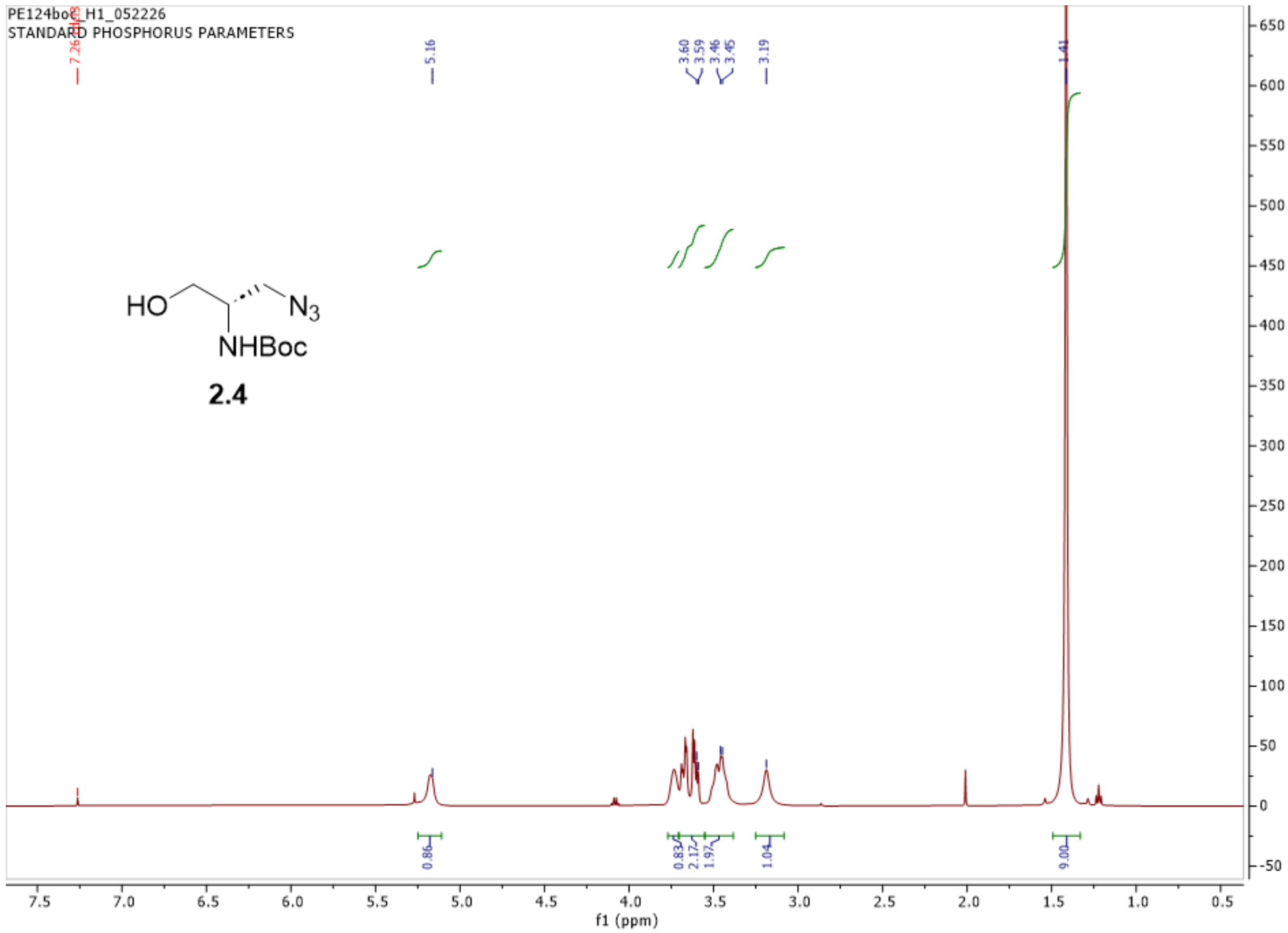
PE124boc_H1_052226
STANDARD PHOSPHORUS PARAMETERS
7.26
5.16
3.60
3.59
3.46
3.45
3.19
1.41
HO
N3
NHBoc
2.4
0.86
0.83
2.17
1.97
1.04
9.00
f1 (ppm)

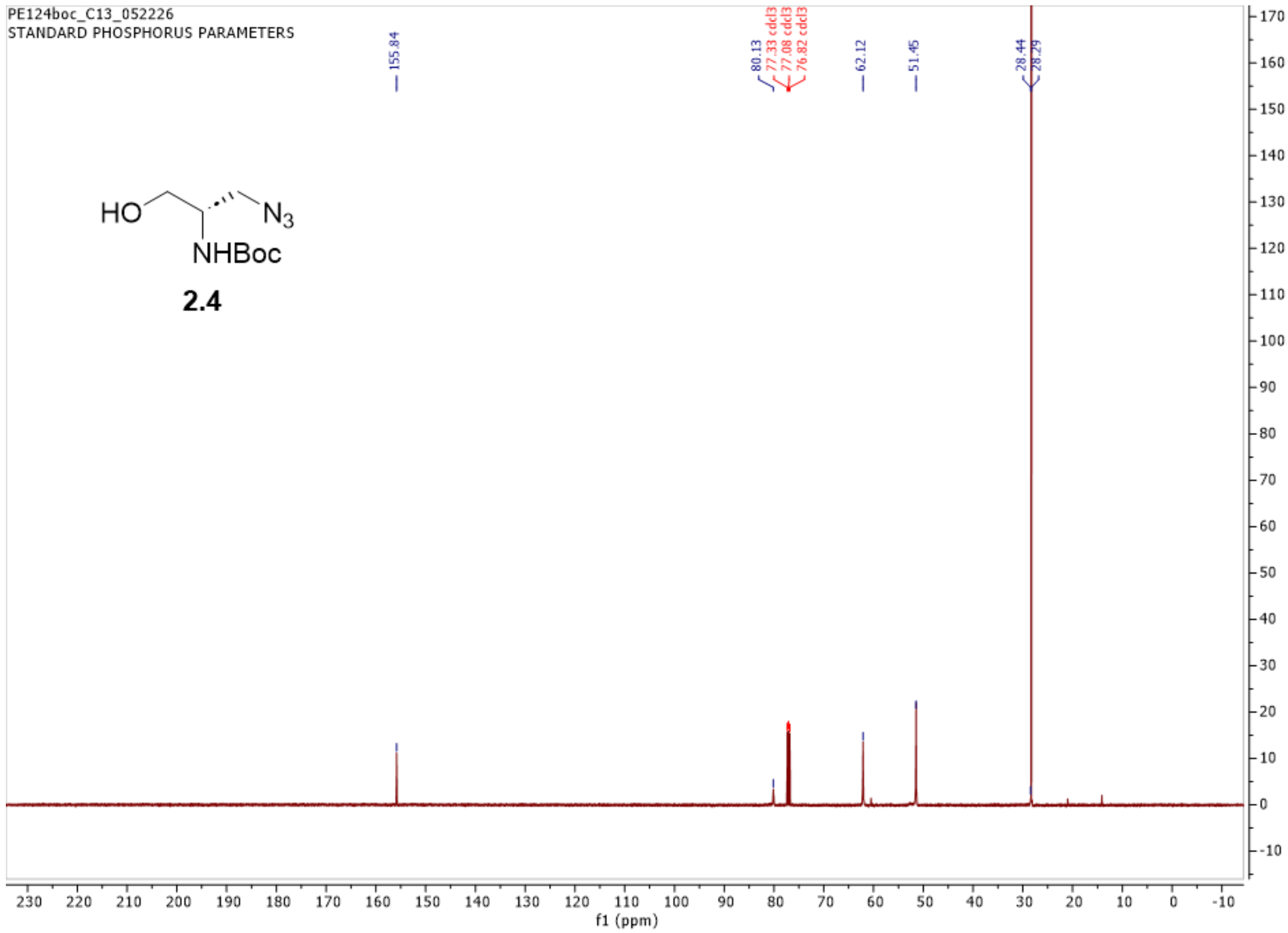
PE124boc_C13_052226
STANDARD PHOSPHORUS PARAMETERS
155.84
80.13
77.33 cdcl3
77.08 cdcl3
76.82 cdcl3
62.12
51.45
28.44
28.29
HO
N3
NHBoc
2.4
f1 (ppm)

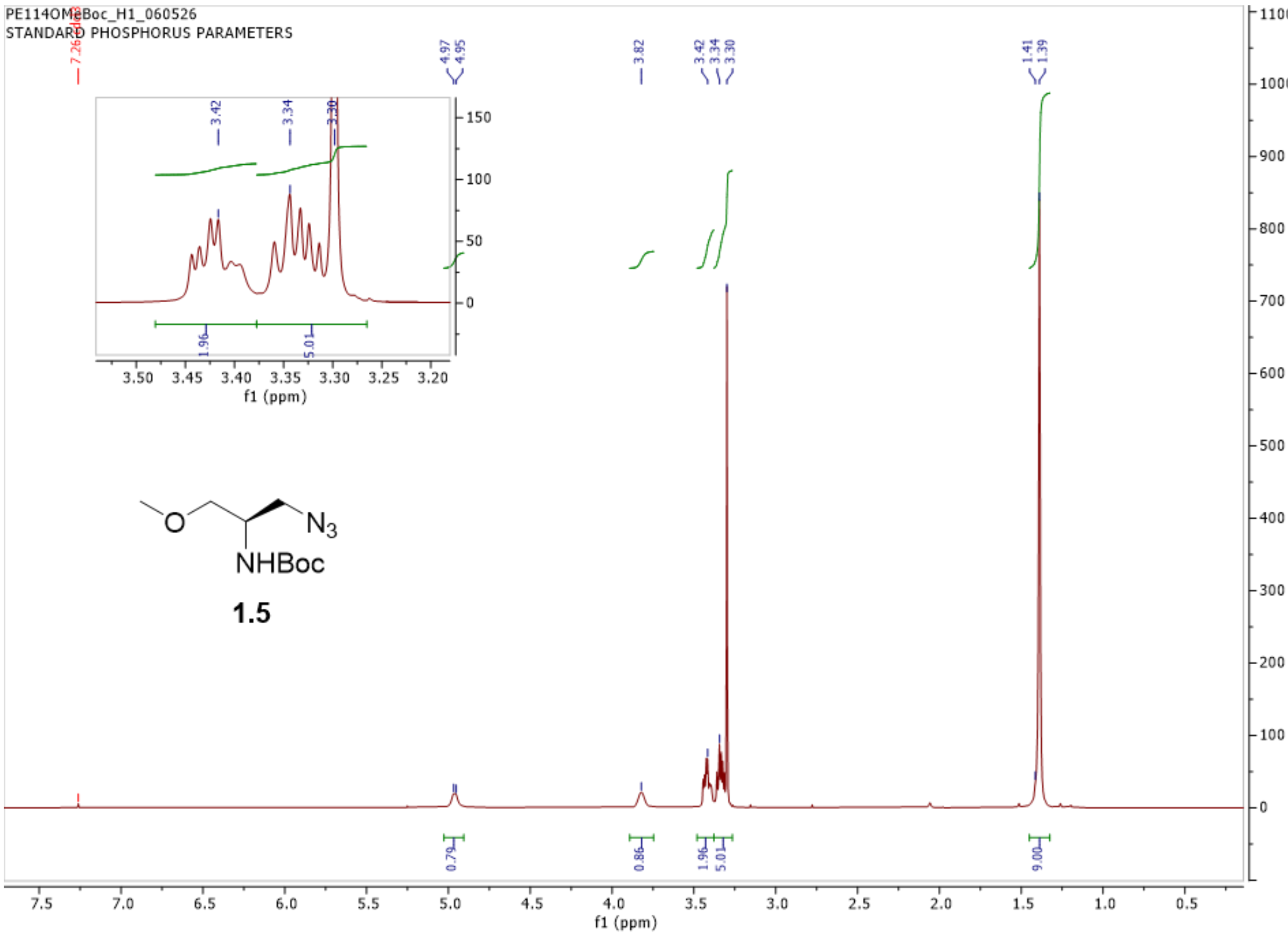
PE114OMeBoc_H1_060526
STANDARD PHOSPHORUS PARAMETERS
7.26
4.97
4.95
3.82
3.42
3.34
3.30
1.41
1.39
3.42
3.34
3.30
1.96
5.01
f1 (ppm)
O
N3
NHBoc
0.79
0.86
1.96
5.01
9.00
f1 (ppm)

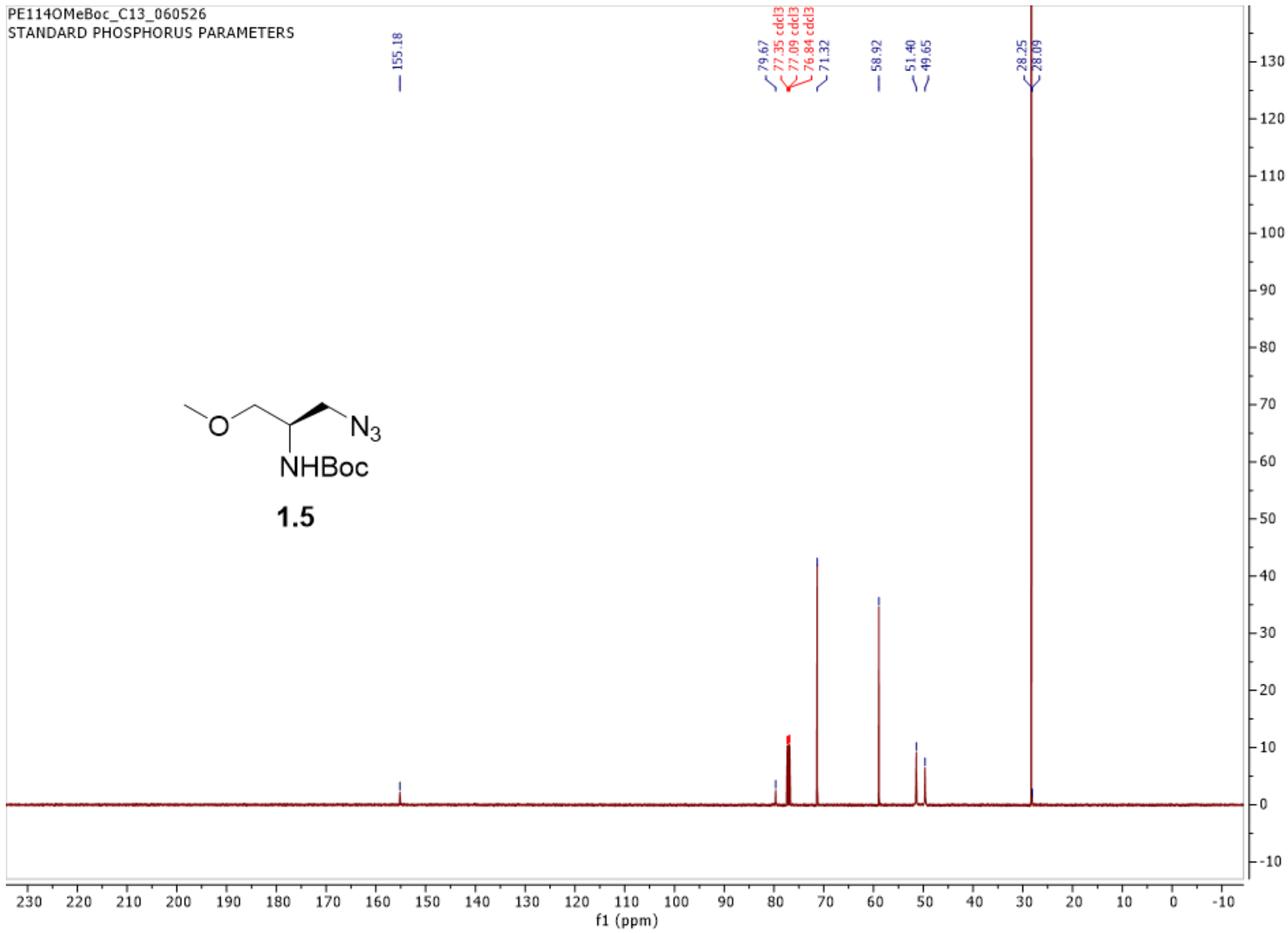
PE114OMeBoc_C13_060526
STANDARD PHOSPHORUS PARAMETERS
155.18
79.67
77.35 cdcl3
77.09 cdcl3
76.84 cdcl3
71.32
58.92
51.40
49.65
28.25
28.09
N3
NHBoc
1.5
f1 (ppm)

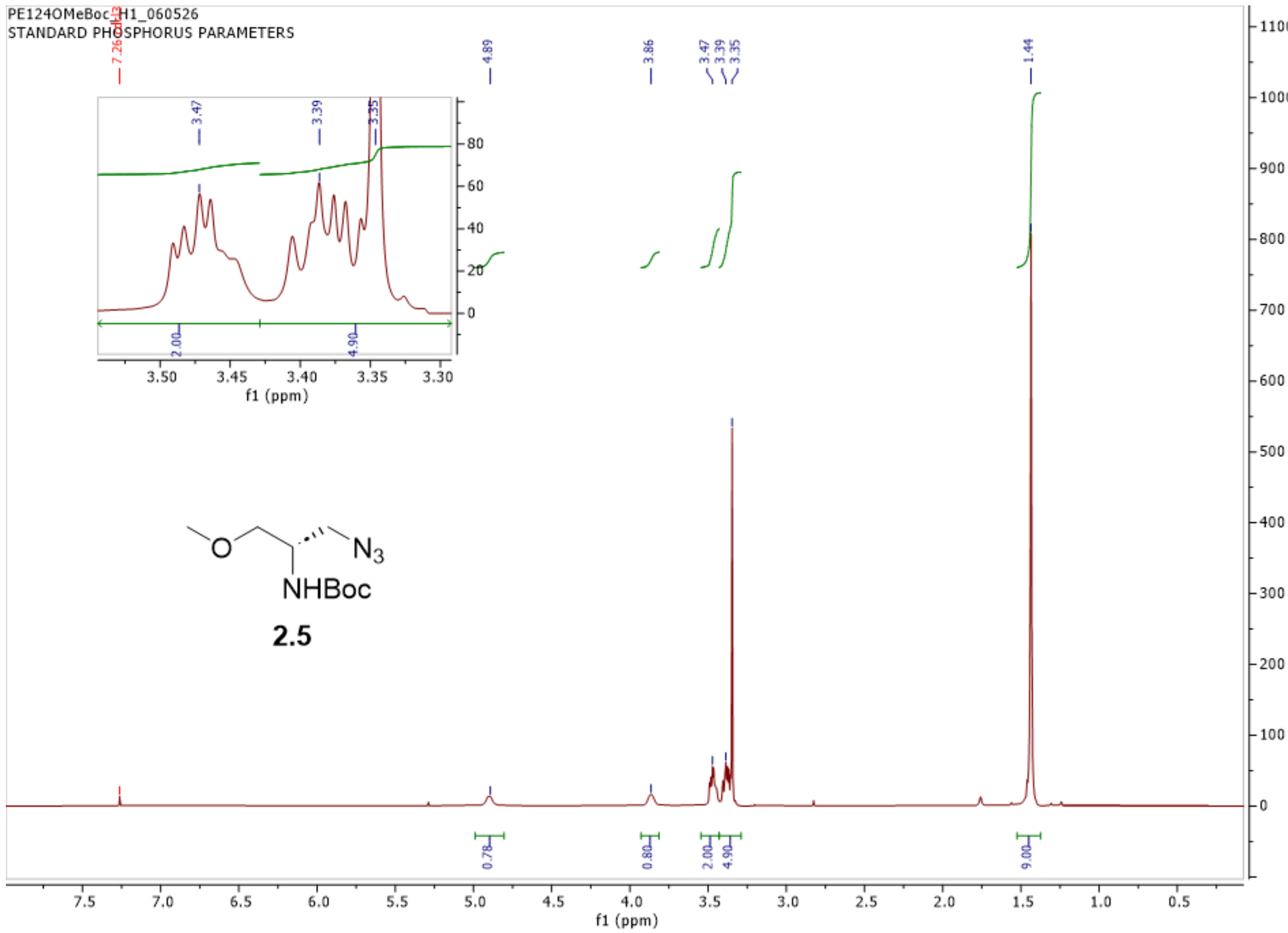
PE124OMeBocH1_060526
STANDARD PHOSPHORUS PARAMETERS
NHBoc
2.5
f1 (ppm)

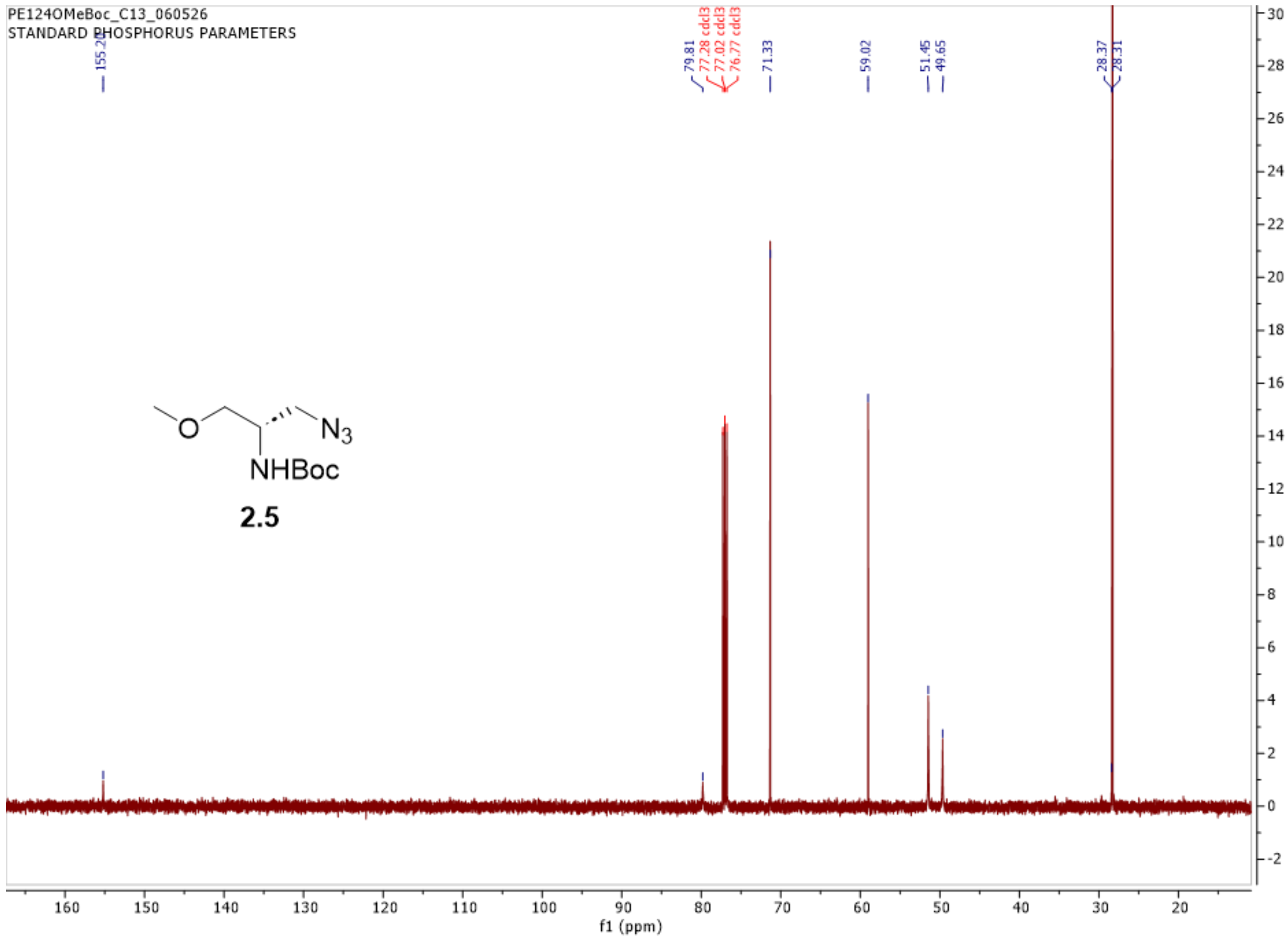
PE124OMeBoc_C13_060526
STANDARD PHOSPHORUS PARAMETERS
155.29
79.81
77.28 cdcl3
77.02 cdcl3
76.77 cdcl3
71.33
59.02
51.45
49.65
28.37
28.31
NHBoc
2.5
f1 (ppm)

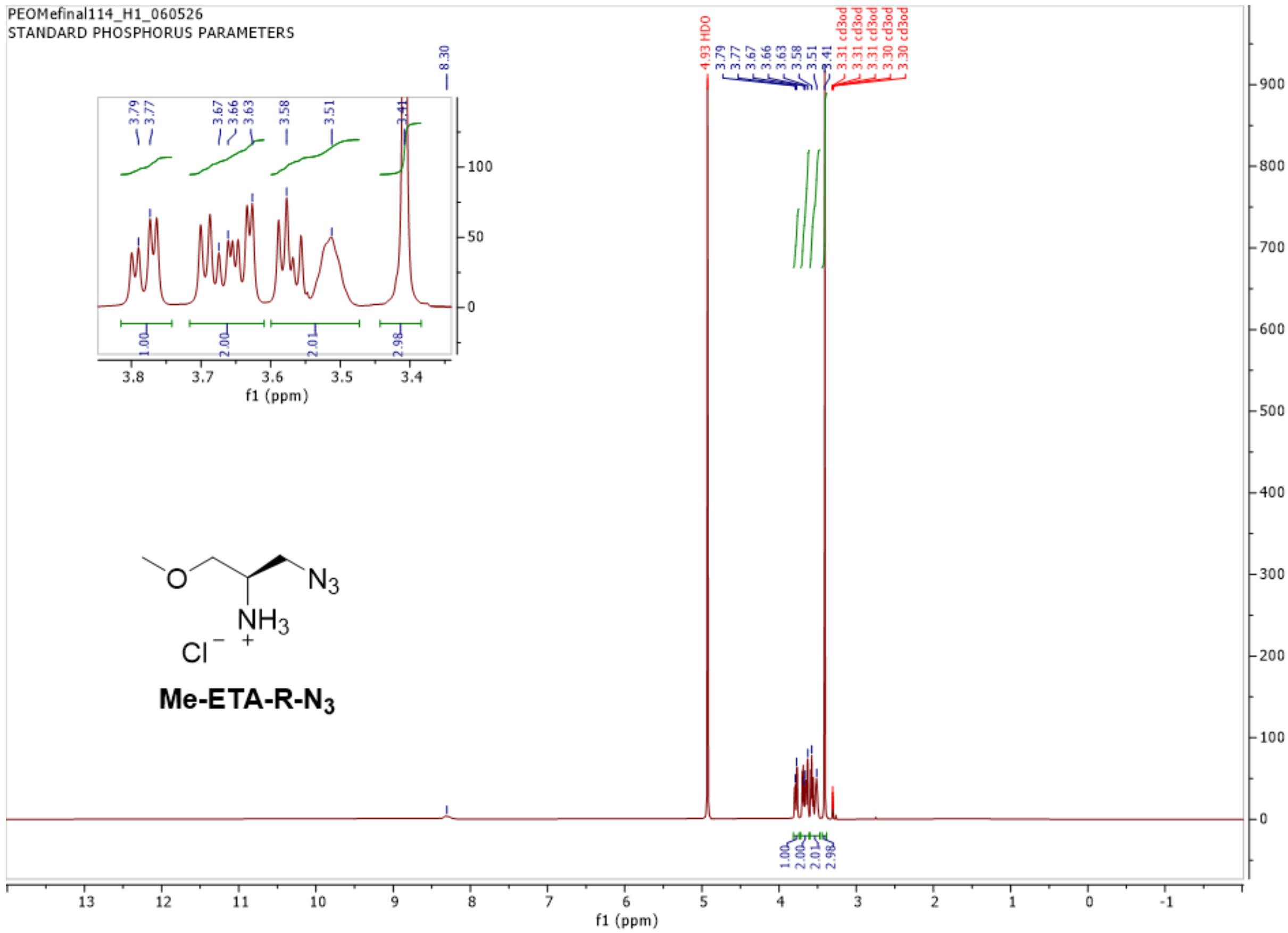
PEOMefinal114_H1_060526
STANDARD PHOSPHORUS PARAMETERS
4.93 HDO
3.79
3.77
3.67
3.66
3.63
3.58
3.51
3.41
3.31 cd3od
3.31 cd3od
3.31 cd3od
3.30 cd3od
3.30 cd3od
8.30
1.00
2.00
2.01
2.98
f1 (ppm)
N3
NH3
Cl
Me-ETA-R-N3

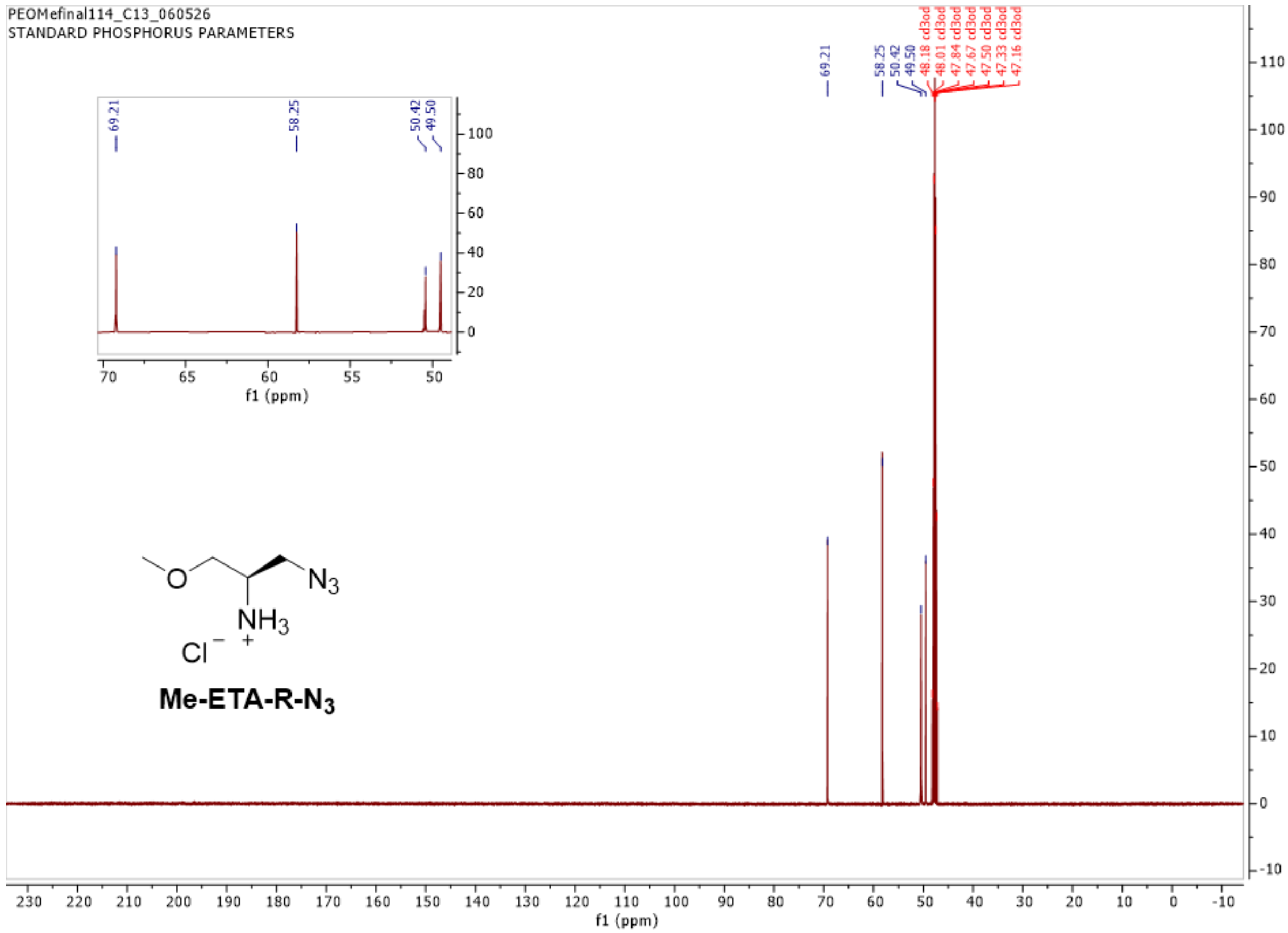
PEOMefinal114_C13_060526
STANDARD PHOSPHORUS PARAMETERS
69.21
58.25
50.42
49.50
48.18 cd3od
48.01 cd3od
47.84 cd3od
47.67 cd3od
47.50 cd3od
47.33 cd3od
47.16 cd3od
f1 (ppm)
N3
NH3
Cl
Me-ETA-R-N3

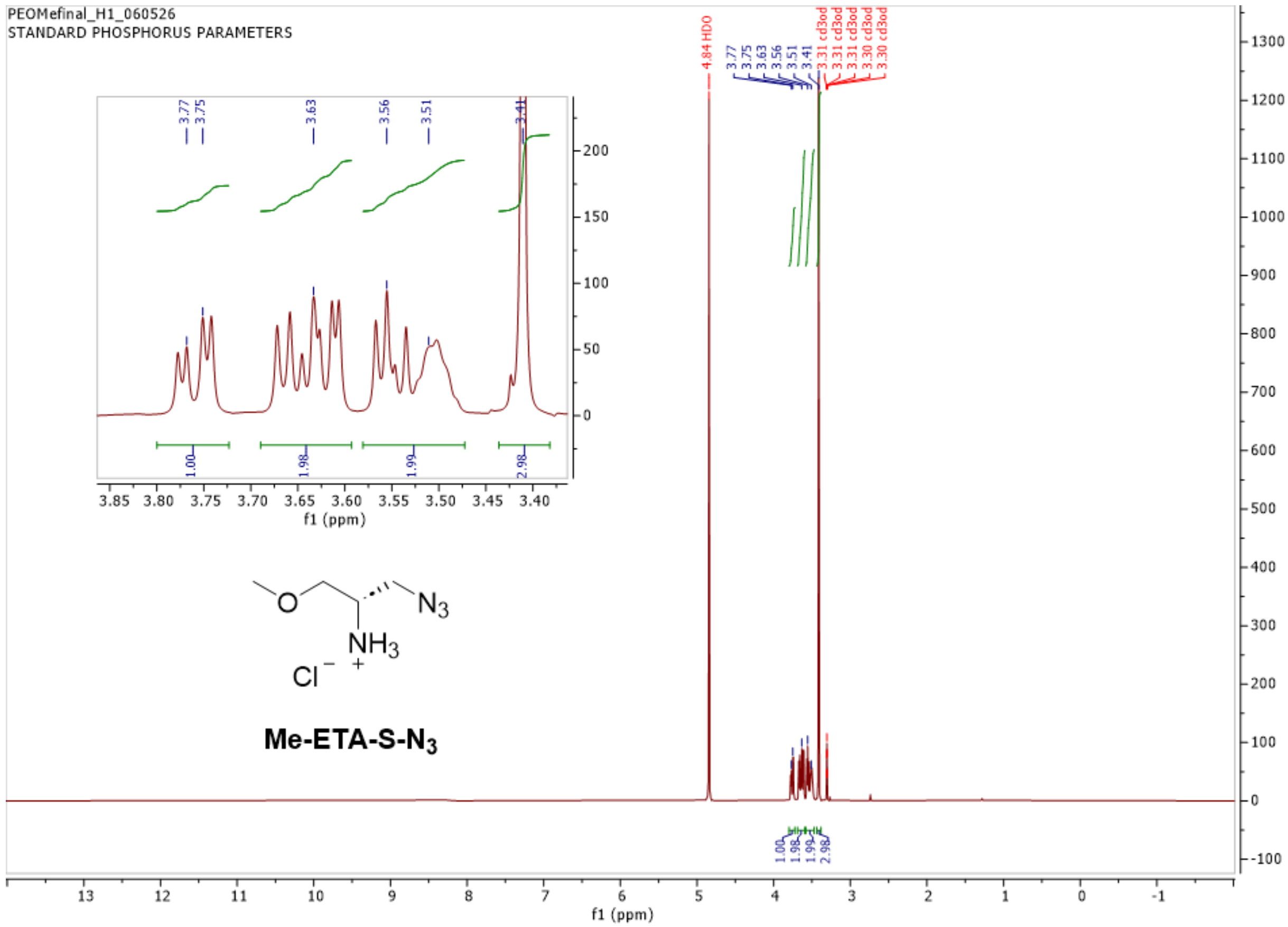
PEOMefinal_H1_060526
STANDARD PHOSPHORUS PARAMETERS
Me-ETA-S-N3
f1 (ppm)

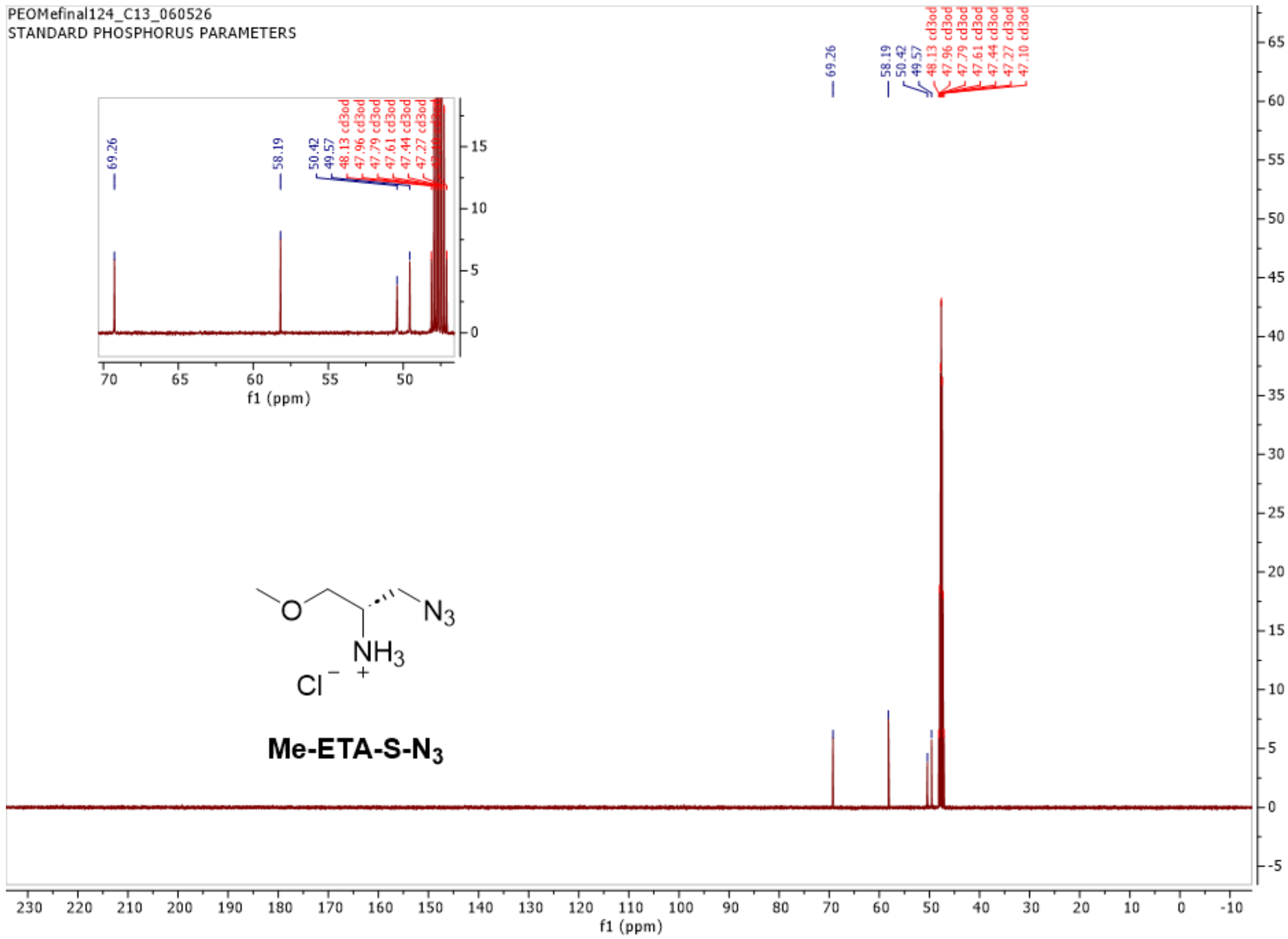
PEOMefinal124_C13_060526
STANDARD PHOSPHORUS PARAMETERS
69.26
58.19
50.42
49.57
48.13 cd3od
47.96 cd3od
47.79 cd3od
47.61 cd3od
47.44 cd3od
47.27 cd3od
47.10 cd3od
f1 (ppm)
Me-ETA-S-N3
